# A linked land-sea modeling framework to inform ridge-to-reef management in high oceanic islands

**DOI:** 10.1371/journal.pone.0193230

**Published:** 2018-03-14

**Authors:** Jade M. S. Delevaux, Robert Whittier, Kostantinos A. Stamoulis, Leah L. Bremer, Stacy Jupiter, Alan M. Friedlander, Matthew Poti, Greg Guannel, Natalie Kurashima, Kawika B. Winter, Robert Toonen, Eric Conklin, Chad Wiggins, Anders Knudby, Whitney Goodell, Kimberly Burnett, Susan Yee, Hla Htun, Kirsten L. L. Oleson, Tracy Wiegner, Tamara Ticktin

**Affiliations:** 1 Department of Natural Resources and Environmental Management, University of Hawaiʻi, Honolulu, Hawaiʻi, United States of America; 2 Hawaiʻi Department of Health, Honolulu, Hawaiʻi, United States of America; 3 Department of Environment and Agriculture, Curtin University, Perth, Australia; 4 Fisheries Ecology Research Lab, University of Hawaiʻi, Honolulu, Hawaiʻi, United States of America; 5 University of Hawaii Economic Research Organization, University of Hawaiʻi, Honolulu, Hawaiʻi, United States of America; 6 University of Hawaiʻi Water Resources Research Center, University of Hawaiʻi, Honolulu, Hawaiʻi, United States of America; 7 Wildlife Conservation Society, Melanesia Program, Suva, Fiji; 8 National Geography Society, Washington, DC, United States of America; 9 CSS, Inc., Fairfax, Virginia, United States of America; 10 NOAA National Centers for Coastal Ocean Science, Silver Spring, Maryland, United States of America; 11 Natural Capital Project, Stanford University, Palo Alto, California, United States of America; 12 Kamehameha Schools Natural and Cultural Resources, Kailua-Kona, Hawaiʻi, United States of America; 13 Limahuli Garden and Preserve, National Tropical Botanical Garden, Hā`ena, Hawaiʻi, United States of America; 14 Hawaiʻi Institute of Marine Biology, University of Hawaiʻi, Honolulu, Hawaiʻi, United States of America; 15 The Nature Conservancy, Hawaii Marine Program, Honolulu, Hawaiʻi, United States of America; 16 Department of Geography, Environment and Geomatics, University of Ottawa, Ottawa, Ontario, Canada; 17 U.S. Environmental Protection Agency, Gulf Ecology Division, Gulf Breeze, Florida, United States of America; 18 Marine Science Department, University of Hawaiʻi, Hilo, Hawaiʻi, United States of America; 19 Department of Botany, University of Hawaiʻi, Honolulu, Hawaiʻi, United States of America; Academia Sinica, TAIWAN

## Abstract

Declining natural resources have led to a cultural renaissance across the Pacific that seeks to revive customary ridge-to-reef management approaches to protect freshwater and restore abundant coral reef fisheries. Effective ridge-to-reef management requires improved understanding of land-sea linkages and decision-support tools to simultaneously evaluate the effects of terrestrial and marine drivers on coral reefs, mediated by anthropogenic activities. Although a few applications have linked the effects of land cover to coral reefs, these are too coarse in resolution to inform watershed-scale management for Pacific Islands. To address this gap, we developed a novel linked land-sea modeling framework based on local data, which coupled groundwater and coral reef models at fine spatial resolution, to determine the effects of terrestrial drivers (groundwater and nutrients), mediated by human activities (land cover/use), and marine drivers (waves, geography, and habitat) on coral reefs. We applied this framework in two ‘ridge-to-reef’ systems (Hā‘ena and Ka‘ūpūlehu) subject to different natural disturbance regimes, located in the Hawaiian Archipelago. Our results indicated that coral reefs in Ka‘ūpūlehu are coral-dominated with many grazers and scrapers due to low rainfall and wave power. While coral reefs in Hā‘ena are dominated by crustose coralline algae with many grazers and less scrapers due to high rainfall and wave power. In general, Ka‘ūpūlehu is more vulnerable to land-based nutrients and coral bleaching than Hā‘ena due to high coral cover and limited dilution and mixing from low rainfall and wave power. However, the shallow and wave sheltered back-reef areas of Hā‘ena, which support high coral cover and act as nursery habitat for fishes, are also vulnerable to land-based nutrients and coral bleaching. Anthropogenic sources of nutrients located upstream from these vulnerable areas are relevant locations for nutrient mitigation, such as cesspool upgrades. In this study, we located coral reefs vulnerable to land-based nutrients and linked them to priority areas to manage sources of human-derived nutrients, thereby demonstrating how this framework can inform place-based ridge-to-reef management.

## Introduction

Over the past century, climate change became a global threat to coral reefs as it directly impacts corals through bleaching, ocean acidification, and intensified storms [1–3]. At the local scale, human activities also impact coral reefs through increasing land-based source pollution and fishing pressure [3–5]. These trends have led some coral reefs to shift towards algae dominated phases, causing the decline of important resources upon which human wellbeing depends [6,7]. Thus, managing for coral reef resilience has become a priority for conservation planning [8]. Resilience is the capacity of an ecosystem to cope with its disturbance regime without shifting to an alternative state, while maintaining its functions and delivery of ecosystem service [9]. Ridge-to-reef management has been widely advocated to foster coral reef resilience, though the degree to which managing local drivers can benefit coral reefs varies among places [10,11]. The types of management actions needed to maintain coral reef resilience will differ spatially, depending on the characteristics of each ridge-to-reef system.

Natural disturbance regimes have shaped the character of coral reef ecosystems over geologic time scales by changing community structures, physical environments, and resource and space availability [12]. Coral reef disturbance regimes consist of a mixture of infrequent events, such as hurricanes, which reduce habitat complexity, and more frequent events, such as waves which dictate coral growth, and freshwater inundation that increases coral mortality [4,13–15]. The structure of coral reef communities (hereafter—coral reefs) further depends on local natural and anthropogenic drivers [16,17]. Fishing is an anthropogenic driver altering the structure of fish populations by removing key functional groups, such as herbivores [18,19], which play an important role in coral reef resilience by controlling the abundance of turf and macroalgae, and freeing space for larval recruitment [16,17,20]. Terrestrial drivers (e.g., nutrients and sediment) and marine drivers (e.g., habitat topography) shape the structure of the benthic community [21–26]. Land cover/use, such as coastal development can be a source of human-derived nutrients that promote algae growth, while agriculture can increase sedimentation and cause coral mortality [26–29]. Coral reef community structure is, therefore, a result of its natural disturbance regime and a combination of local natural and anthropogenic drivers.

Community-based movements across the Pacific seek to restore customary resource management systems that recognize the importance of land and sea connections to promote social and ecological resilience, such as the *ahupua‘a* (ridge-to-reef) system in Hawai‘i and the concept of *vanua* in Fiji [30–33]. High Pacific islands are ideal models to study the effects of land-sea connections on coral reefs under various natural disturbance regimes [34]. As a result of their small size and steep elevational gradients, land and sea are tightly connected through anthropogenic and natural processes [35]. Due to their volcanic origin, island age can range from zero (actively growing) to millions of years old, and thus represent different stages of erosion [36]. The trade winds combined with rain shadows from volcanic peaks result in a gradient of rainfall, with wet windward and dry leeward exposures [37,38]. Owing to their location in the Pacific ocean, wave power impacts on all shorelines under seasonal patterns governed by distant storms [39,40]. Using traditional ridge-to-reef conceptual frameworks can help us understand these systems and support the restoration of community-based management in Pacific Islands.

To manage for coral reefs resilience, decision-makers need to understand the effects of both natural and anthropogenic drivers, from ridge-to-reef [24,41]. To assess the impacts and recovery of coral reefs subject to multiple drivers, researchers have generally relied on long-term quantitative measurements, which are rare, costly, and typically conducted at limited spatial scales [42–45]. Therefore, ecological modeling is a useful tool to foster understanding of coral reefs under co-occurring drivers and inform management at relevant spatial scales [11,46]. Although a variety of models have recently been developed to explore the influence of natural and anthropogenic drivers on coral reefs, only a few have incorporated land-sea connections and found that it changed conservation priorities [27,47–54]. However, these applications remain too coarse (1 km resolution) to support watershed-scale management for high Pacific Islands.

To address this gap, we built a novel linked land-sea modeling framework at a fine spatial scale, based on local data. We applied this framework in two *ahupua‘a* (Hā‘ena and Ka‘ūpūlehu), with different natural disturbance regimes, located at opposite ends of the main Hawaiian Islands, to compare outcomes and inform place-based ridge-to-reef management. Although both communities have recently implemented marine reserves to actively limit fishing impacts on coral reefs in each place [55–57], we developed this framework to determine the effects of land-sea connections and identify terrestrial management actions that could promote coral reef resilience. Land-sea connections can take multiple pathways, which range from streams and storm water runoff, to groundwater discharge [15,55–57]. Though less studied, groundwater has been found in many instances to exceed surface runoff and be a primary vector for land-based nutrients to coral reefs [58–61].

Ka‘ūpūlehu is located in a very dry region, lacks perennial streams, and surface runoff is uncommon. Submarine groundwater discharge (SGD) was found to be the primary vector of nutrients to coastal waters in this area [59]. Although Hā‘ena is located in a wet region, where surface water discharge largely exceeds SGD, Knee et al. [62] showed that SGD nutrient flux are significantly greater than surface water, and account for over 70% of the total coastal nutrient discharge. Because there is little agriculture and SGD is the primary vector for land-based nutrients in both *ahupua‘a* [60,62], we linked land and sea through nutrient enriched groundwater. We, then, modeled coral reef benthic and fish indicators, derived from ecological surveys, as a function of freshwater and nutrient flux from groundwater, and important marine drivers, including waves, local geography, and habitat [22,25,63]. By calibrating this framework separately for Hā‘ena and Kaʻūpūlehu, we gained insights on the relative effects of terrestrial and marine drivers on coral reefs subject to different natural disturbance regimes and examined the following research questions: (1) Where are the highest human-derived nutrient flux in each *ahupua‘a*? (2) What are the drivers differentiating these two coral reef systems? (3) How are coral reefs shaped by these drivers in each place? (4) Where land-based management actions could promote resilience of these coral reefs in a changing climate?

## Methods

### Site descriptions

**Fig 1:**
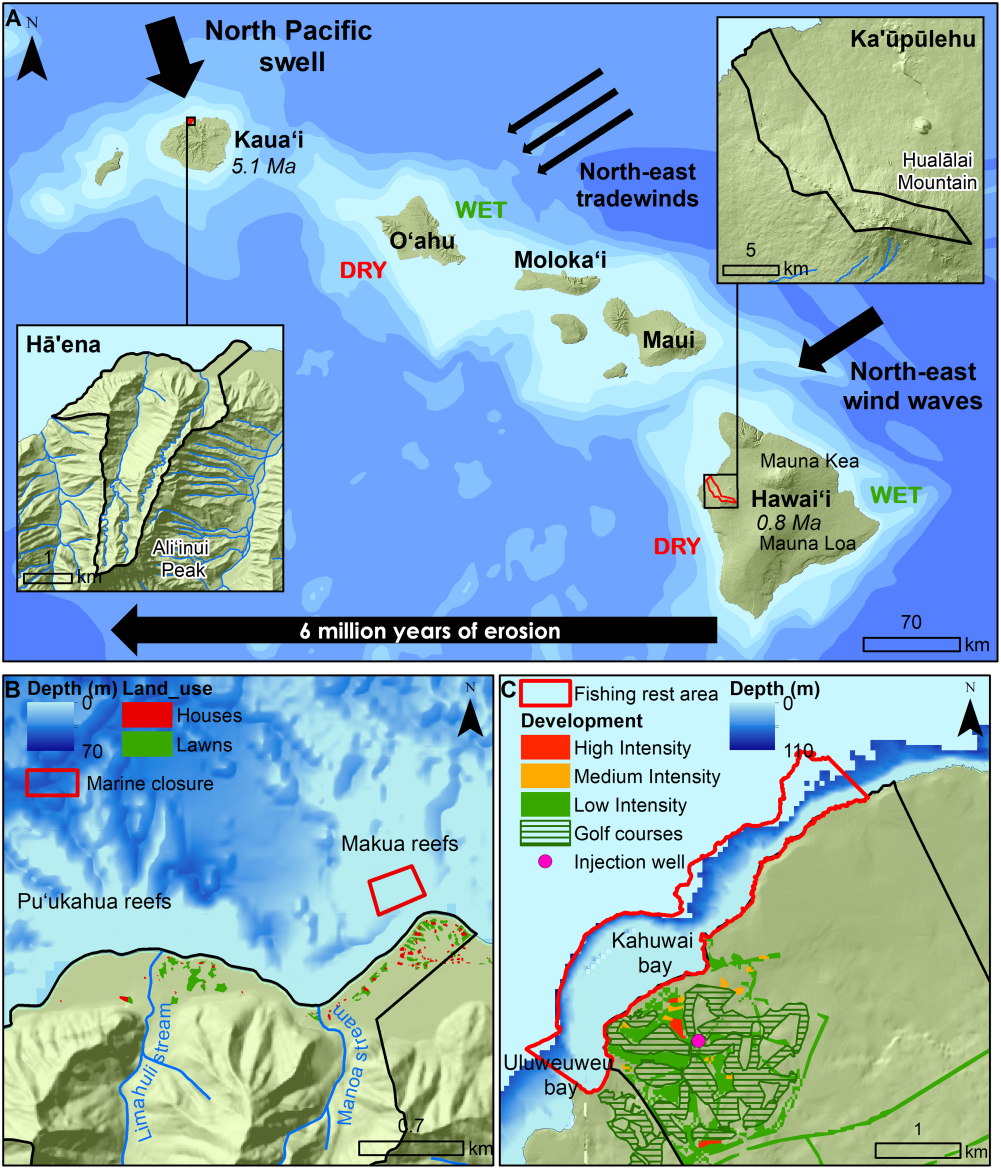
Study sites location. (A) Location of study sites on Kauaʻi and Hawaiʻi along the main Hawaiian Island chain, with island age and the direction of the prevailing north-east tradewinds and ocean swell indicated. Land use/cover and marine closure/fishing rest area are shown for (B) Hā‘ena and (C) Ka‘ūpūlehu.

This research focused on two *ahupuaʻa* at the opposite ends of the main Hawaiian Islands. Hā‘ena is located on the windward side of Kauaʻi Island and Ka‘ūpūlehu is located on the leeward side of Hawai‘i Island (Fig 1A) (further described in Table 1). Geologically older and exposed to the trade winds, Hā‘ena receives high rainfall, resulting in steeply eroded cliffs, with high fluvial and groundwater inputs [64]. Geologically younger and located in the rain shadows of Mauna Loa and Mauna Kea mountains, Ka‘ūpūlehu is very dry and minimally eroded, resulting in poorly developed ephemeral stream channels and large SGD [55,60]. At Hā‘ena, fishing pressure was relatively lower than Ka‘ūpūlehu prior to the establishment of the marine closures (Fig 1C) [65].

**Table 1:** Study site attributes.

Attributes	Hā‘ena	Ka‘ūpūlehu
Island age (Ma)	5.1	0.8
Ahupua‘a size (km^2^)	7.3	104
Maximum elevation (m)	1,006 (Aliʻinui Peak)	2,518 (Hualālai Mountain)
Annual rainfall (mm.yr^-1^)	High (4,040)	Low (1,350 to 260)
Perennial streams	2	0
Coastline length (km)	4	7.4
Reef area (km^2^)	7.6	3.2
Dominant benthic substrate	Crustose coralline algae (CCA)	Coral
Mean total resource fish biomass (g.m^-2^)	7.35	4.53
Coastal development	136 private residences	193 private residences 2 large luxury resorts 1 golf course
Management regime (year established)	Community Based Subsistence Fisheries Management Area (2016)	Community-led 10-year fishing rest period (2016)
Key land owners	State of Hawaiʻi A non-profit organization (National Tropical Botanical Garden)	Private land owner (Kamehameha Schools)

### Overview of the linked land-sea modeling framework

**Fig 2:**
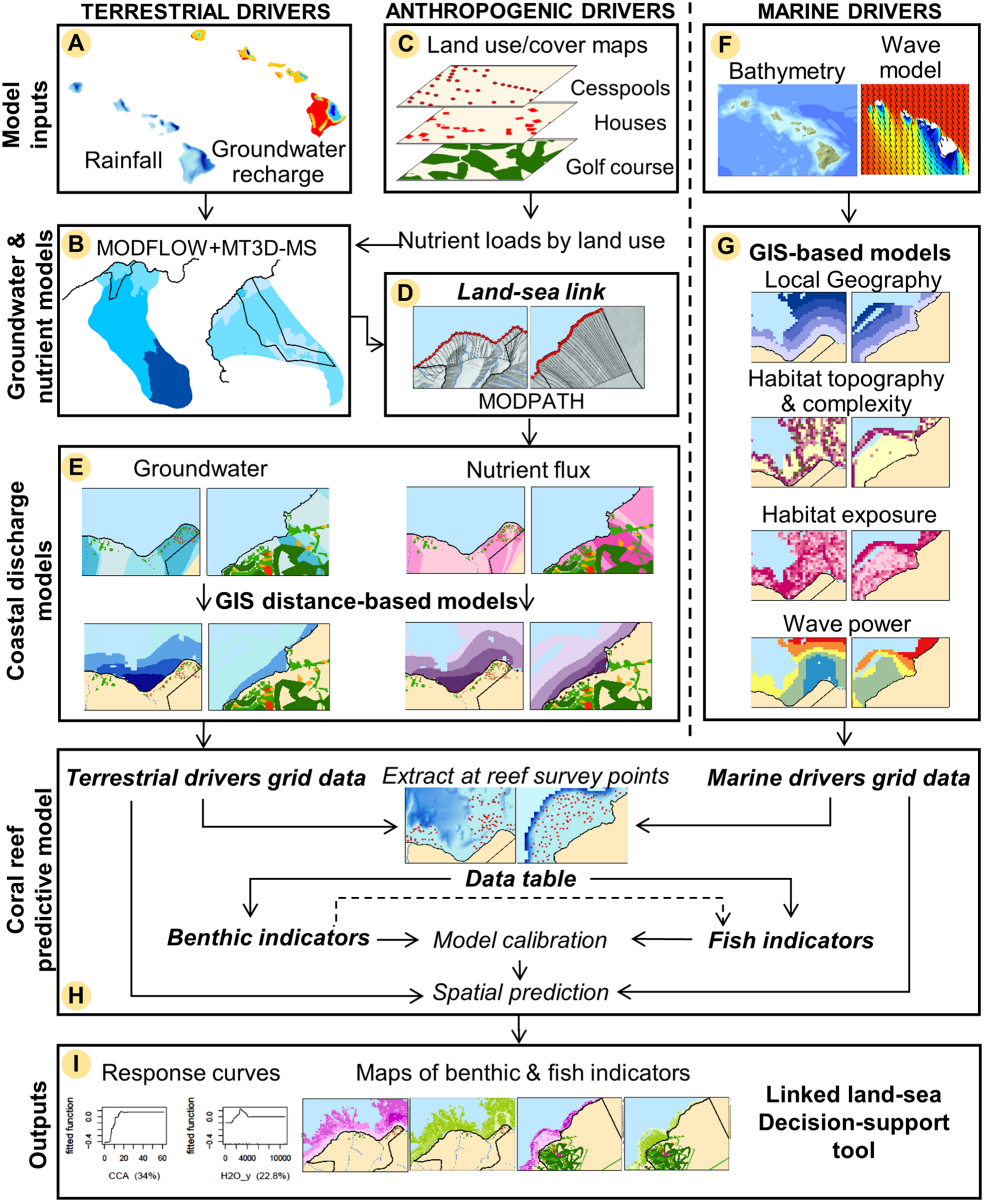
Linked land-sea modeling framework. Based on (A) climate, groundwater recharge, and recharge nutrient concentration data, (B) groundwater flow (m^3^.yr^-1^) and nutrients concentrations (mg.L^-1^) were modeled using MODFLOW and MT3D-MS. (C) Nutrient flux (kg.yr^-1^) from anthropogenic drivers were added to the background nutrient flux. (D) A land-sea link was created using MODPATH. (E) The coastal discharge models used the groundwater flow (m^3^.yr^-1^) and nutrients flux (kg.yr^-1^) and GIS distance-based models to generate the terrestrial driver grid data. (F) The SWAN wave model and bathymetry data were coupled with (G) GIS-based models to generate the marine driver grid data. (H) The coral reef predictive models were calibrated on coral reef survey data. (I) Outputs were: (1) response curves, (2) maps of benthic (% cover) and fish (g.m^-2^) indicators, and (3) a linked land-sea decision-support tool. (The wave model image in panel G is reprinted from [66] under a CC BY license, with permission from Charles Fletcher, original copyright 2009. Refer to S1 File).

Our modeling framework linked land cover/use to coral reefs through nutrient-enriched groundwater flux, using spatially-explicit groundwater and coral reef predictive models calibrated with existing empirical and remote sensing data (Fig 2). Based on climate, groundwater recharge, and recharge nutrient concentration data, groundwater flow (m^3^.yr^-1^) and nutrient flux (kg.yr^-1^) discharging at the coast were modeled using MODFLOW and MT3D-MS at Hā‘ena (15x15 m) and Ka‘ūpūlehu (50x50 m). Spatially explicit nutrient flux (kg.yr^-1^) from land cover/use were added to the groundwater background nutrient flux. A land-sea link was created by sub-dividing the groundwater model domain into ‘flow tubes’ (~200 m width) ending at pour points along the shoreline using MODPATH. To quantify the different effects of freshwater and nutrient discharge on coral reefs, we computed the total groundwater flow (m^3^.yr^-1^) and nutrient flux (kg.yr^-1^) for each flow tube using ZONEBUDGET and diffused those values from each pour point into the coastal zone using GIS distance-based models to generate the terrestrial driver grid data (60x60 m). The SWAN wave model and bathymetry data were coupled with GIS-based models, to generate marine driver grid data (geography, habitat, and waves) (60x60 m). The coral reef predictive models were Boosted Regression Trees (BRT) calibrated on local coral reef survey data, which generated response curves representing the relationships of each individual driver to each coral reef benthic and fish indicator and predicted maps of benthic (% cover) and fish (g.m^-2^) indicators (60x60 m). Once calibrated on local data, this linked land-sea framework can be used as a decision-support tool to identify priority areas for nutrient mitigation.

### Coral reef indicators and field data

**Fig 3:**
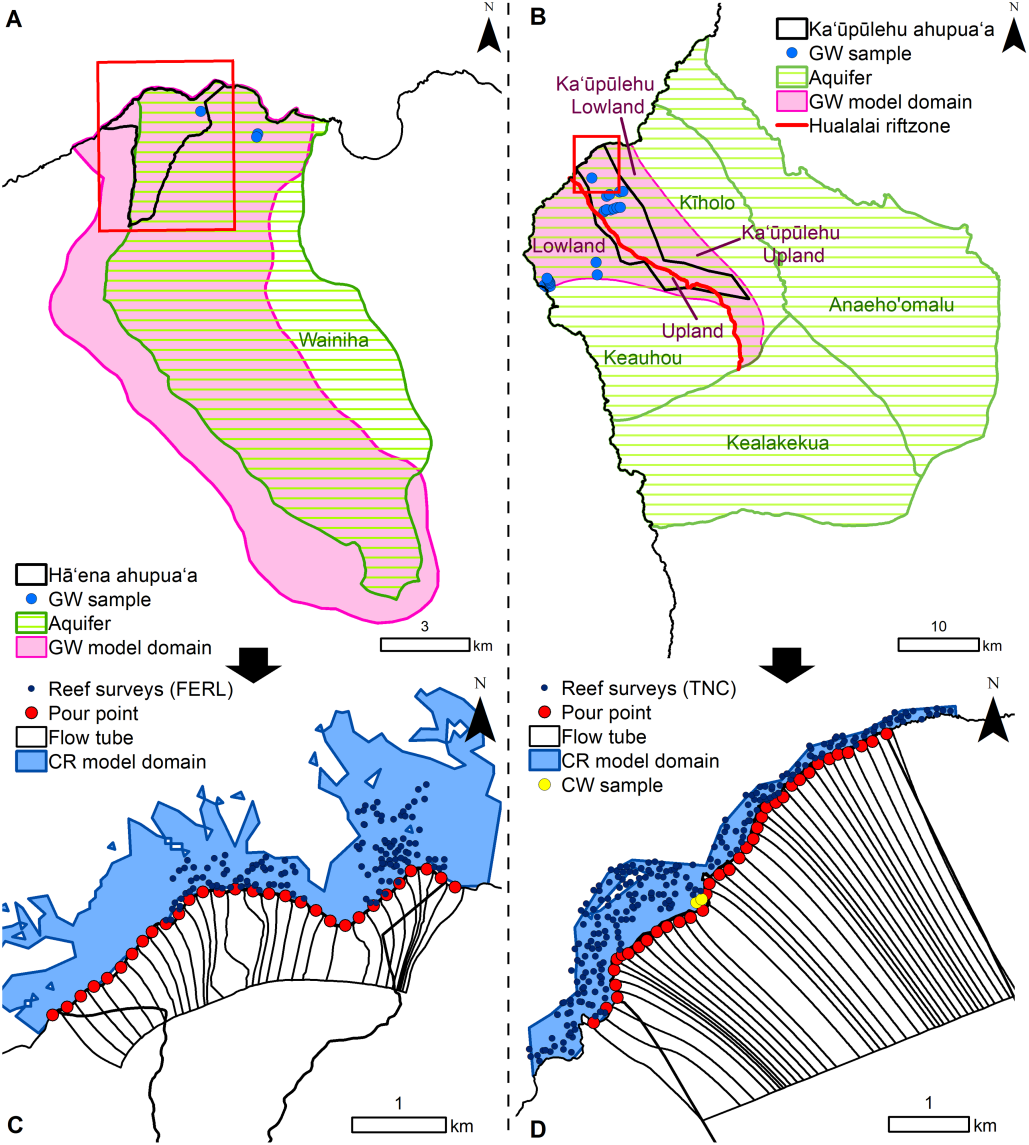
Model domains and land-sea link. The groundwater (GW) model domain at (A) Hā‘ena (15x15 m) overlaps with the Wainiha Aquifer. The GW model domain at (B) Ka‘ūpūlehu (50x50 m) was divided into 4 zones (Keauhou upland and lowland, Ka‘ūpūlehu upland and lowland) and spreads across the Kīholo and Keauhou Aquifers and bisected by a rift line. In the coastal zone, the groundwater model domain was sub-divided into narrow (~200 m) flow tubes ending at pour points along the shoreline to spatially link the groundwater model outputs to SGD. Reef surveys were provided by FERL at (C) Hā‘ena and TNC at (D) Ka‘ūpūlehu. Based on the depth of coral reef surveys, the coral reef (CR) model domain (60x60 m) extended from the shoreline to –15 m at Hā‘ena and –22 m at Ka‘ūpūlehu.

To measure ecological resilience, we considered the abundance of four benthic groups (% cover) and the biomass of four fish groups (g.m^-2^) based on their ecological roles and cultural importance to Native Hawaiians [20,67]. The benthic indicators included calcifying organisms (CCA and scleractinian corals) and benthic algae (turf and macroalgae) (see S1 Table for more details). Resource fishes identified as important for subsistence and cultural practices by Indigenous Hawaiian communities (e.g., Acanthuridae, Scaridae, Carangidae) were modeled according to their ecological role: (1) browsers, (2) grazers, (3) scrapers, and (4) piscivores (see S1 and S2 Tables for more details) [20,68–70]. We derived percent cover of the benthic indicators and biomass of the fish indicators (g.m^-2^) from reef survey data collected by the Fisheries Ecology Research Lab (FERL) at the University of Hawaiʻi and The Hawaiʻi Nature Conservancy (TNC) reef monitoring program (Fig 3). At Hā‘ena, the field dataset comprised 126 survey locations randomly stratified by habitat (nearshore, back-reef, and fore-reef areas) and allocated proportionately across Makua and Puʻukahua reef complex (Fig 3C), collected over two sampling periods, July 2013 and August 2014 (refer to [71] for more details). At Ka‘ūpūlehu, the field dataset comprised 243 survey locations randomly stratified across two factors: management status (inside and outside the Fisheries Replenishment Area) and reef types, collected over two sampling periods, 2012 (N = 166) and 2013 (N = 78) (Fig 3D) (refer to [72] for more details).

### Groundwater models

We assigned the boundary conditions of the groundwater models using MODPATH [73,74] with: (1) a flux representing the groundwater recharge at the upslope boundary; (2) no-flow condition at the lateral boundaries; and (3) the elevation of the groundwater head at the coast (layer 1) and submarine (layer 2) boundaries, based on the greater density of seawater compared to freshwater. The groundwater model domains at both sites were set to comprise the groundwater flow path from zones of recharge to coastal discharge, while spanning the entire *ahupua‘a* boundaries and the coastal development. At Hā‘ena, the groundwater model boundaries were aligned with the groundwater divides, which follow the watershed boundaries [75]. Therefore, the model domain was 6,975 ha and spanned four watersheds with perennial streams (i.e., Wainiha River [6,130 ha], Mānoa [253 ha], Limahuli [480 ha] and the Mauna Pūloʻu [112 ha] watersheds) (Fig 3A). At Kaʻūpūlehu, Engott (2011) showed that the majority of the groundwater discharging at the coastline is recharged on the upper slopes of Hualālai Mountain. Using MODPATH [74] in the reverse tracking mode, we traced the groundwater flow lines from the coastline to the upper slopes of Hualālai Mountain to delineate the Aquifer boundaries and define the zone of groundwater recharge based on the convergence of the lines. Consequently, the model domain at Kaʻūpūlehu was 33,400 ha and comprised most of the north-central and central part of the Hualālai Aquifer Sector and assumed no inter-aquifer flow between the Kīholo Aquifer and the Keauhou Aquifer, due to a rift zone bisecting the modeled area (Fig 3B). Given the different size of the model domains, we modeled Hā‘ena at 15x15 m resolution and Kaʻūpūlehu at 50x50 m resolution to maintain computer efficiency.

#### Groundwater flow

We estimated the coastal groundwater discharge (m^3^.yr^-1^) using the groundwater model MODFLOW [76] and applying Eq 1 [55] at each grid cell across the model domain of both sites (Fig 2A & 2B):

ΔGW=R+Inj−ET−Str−Q−Cstl (1)

where Δ*GW* = change in groundwater volume (set to zero under steady state modeling), *R* = groundwater recharge (derived from Eq 2 for Hā‘ena [77] [see below] and from the comprehensive Hawai‘i Island groundwater recharge assessment at >20 m^2^ resolution for Ka‘ūpūlehu [55,78]), *Inj* = water injection volume into the aquifer (set to zero at Hā‘ena and derived from [79] at Ka‘ūpūlehu), *ET* = evapotranspiration from the aquifer (set to zero because both model domains were deeper than the maximum evapotranspiration depth [1.5 m] [78]), *Str* = groundwater discharge to streams. (At Hā‘ena, we estimated at 0.26 m^3^.s^-1^ discharge of groundwater to Wainiha River using gauged flow data [from 2007 to present] [80] and a flow frequency distribution curve [81], then we scaled down the Wainiha River baseflow according to the relative watershed area of the other streams. At Ka‘ūpūlehu, it was set to zero due to lack of perennial streams), *Q* = groundwater withdrawal rate (derived from [82] for both sites), *Cstl* = coastal groundwater discharge (computed as residuals).

A coarse resolution comprehensive recharge assessment was available for the Wainiha Aquifer [77]. To enhance the spatial resolution, we calculated the groundwater recharge (*R*) for Hā‘ena by applying Eq 2 [77] at each grid cell of the model domain, which was modified to account for the leaching effluent from Onsite Sewage Disposal Systems (OSDS) into the groundwater:

R=P+I+OSDS−DR−AE−ΔSS (2)

where *P* = precipitation (derived from a statewide rainfall map at 250 m resolution [37]), *I* = irrigation (set to zero due to the lack of agriculture in the model domain), *OSDS* = leaching effluent from OSDS (refer to section ‘Modeling anthropogenic drivers’), *DR* = direct runoff (assumed at 54% of rainfall [55,77]), *AE* = actual evapotranspiration (derived from a statewide evapotranspiration map at 250 m resolution [38]), and Δ*SS* = change in soil moisture storage (assumed to average out to zero over long term). Our calculated groundwater recharge was within 1% of the Wainiha Aquifer water budget model [77].

#### Groundwater nutrient concentrations

We estimated the dissolved inorganic nitrogen and phosphorus (hereafter—N and P) concentrations (mg.L^-1^) using the nutrient transport model Modular Three-Dimensional Multispecies Transport Model (MT3D-MS) [83] (Fig 2B). In the absence of plant uptake at both sites, N was treated as a conservative transport species, which did not bind to soil or alter to another chemical state [84,85]. Conversely, P binds to most soils, so P concentrations reflected the leachable fraction available to the groundwater [86,87]. Given that P beneath the soil zone cannot bind with soils, we assumed no sorption for wastewater from injection wells and cesspool discharge [88]. The dispersal distance of dissolved nutrients depends on the aquifer heterogeneity, groundwater flow velocity, and molecular diffusion, and was set to 20 m according to local studies [75,88,89].

We assigned representative nutrient concentrations to the groundwater recharge based on concentrations measured in the groundwater within our model domains, thereby indirectly accounting for the biogeochemical reactions in the soil and other horizons. At Hā‘ena, the background nutrient concentrations were derived from samples collected in the Wainiha Aquifer (Fig 3A) [62,90]. Given the limited groundwater samples available at Hā‘ena, background nutrient concentrations were uniformly distributed across the model domain (Table 2). At Ka‘ūpūlehu, the background nutrient concentrations in the Hualālai Aquifer were spatially variable, partly due to the rift zone; therefore, the model domain was divided into four zones (Ka‘ūpūlehu upland and lowland, Keauhou upland and lowland) with their respective nutrients concentrations (Table 2 & Fig 3B). The nutrient concentrations for each zone were derived from Fackrell [91], who sampled 42 locations across the model domain (Fig 3B). We evaluated our modeled nutrient concentrations against these measured nutrient concentrations using linear regression (R^2^ and p-value).

**Table 2:** Annual nutrient concentrations and flux of the groundwater background and land cover/use zones.

Sources	Zones	[N] (mg.L^-1^)	[P] (mg.L^-1^)	N flux (kg.yr^-1^)	P flux (kg.yr^-1^)	Source
Natural (background)	Hā‘ena	0.50	0.20	7.51.ha^-1^	3.00.ha^-1^	[62,90]
	Ka‘ūpūlehu Upland	2.70	0.20	8.55.ha^-1^	0.63.ha^-1^	[55,91]
	Ka‘ūpūlehu Lowland	0.25	0.10	0.65.ha^-1^	0.26.ha^-1^	[55,91]
	Keauhou Upland	1.20	0.15	3.11.ha^-1^	0.26.ha^-1^	[55,91]
	Keauhou Lowland	0.25	0.1	0.72.ha^-1^	0.29.ha^-1^	[55,91]
Anthropogenic	Cesspool^a^^,^^b^	87	19	38	8.3	[92–95]
	Septic system^a^^,^^c^	34.2	1.2	14.9	5.2	[93,96]
	Injection well^d^	5.25	6.8	843	1300	[79]
	Lawn^e^	0.20	0.01	4.5.ha^-1^	0.2.ha^-1^	[97]
	Golf course^f^	7.59	0.54	49.ha^-1^	13.5.ha^-1^	[98]

Groundwater zones were assigned N and P recharge concentrations with the corresponding flux (i.e., concentration x annual recharge).

^a^ Each land parcel assumed a residential unit with three bedrooms at an occupancy rate of 1.5 persons per bedroom generating 435 m^3^.yr^-1^ of wastewater [93].

^b^ The nutrient loading rates were based on sampling conducted on Maui [94].

^c^ The nutrient loading rates were based sampling conducted on Hawai‘i Island [96].

^d^ Wastewater discharge is 160,600 m^3^.yr^-1^ according to the State of Hawaii Injection Permit database [79].

^e^ Assuming a recharge rate of 50 m^3^.ha.d^-1^ + background concentrations [97]

^f^ Golf course fertilization rates were assumed at 879 kg.ha^-1^ of N and 122 kg.ha^-1^ of P, and a leaching rate of 5% for both nutrients [98].

#### Human derived nutrients

To assess how coastal land cover/use influences coral reefs, we quantified N and P recharge concentrations (mg.L^-1^) from coastal development in each *ahupua‘a* and converted those to N and P flux (kg.yr^-1^). In order to do so, we used vector maps, the houses were represented with points and the fertilized green spaces were represented by polygons delineated using aerial photos with a minimum mapping unit of 20 m^2^ (Fig 2C). We then determined the wastewater treatment systems associated with each house using a statewide OSDS database [97]. These were either cesspool or septic tank systems, which both discharge effluent into the groundwater beneath the OSDS location. At Hā‘ena, we estimated a total of 136 houses (99 cesspools; 37 septic systems) and 6 ha of lawn in the model domain [93]. At Ka‘ūpūlehu, we estimated 193 houses with 45 ha of lawn, two resorts disposing of their wastewater after secondary treatment through an injection well, and a golf course (190 ha) [79]. The coastal nutrient flux from lawns and golf courses were based on assumed irrigation and fertilization rates [97,99]. To reflect the best management practices of the Ka‘ūpūlehu community, we used lower fertilization rates on the golf course compared to urbanized areas, like West Maui [100]. We assigned N and P flux to each land cover/use derived from local data where possible, and from literature values, when local data were not available (*see* Table 2 for more details). We used the Groundwater Modeling System (GMS) interface [101] to compute the nutrient loads from the vector-based land cover maps (e.g. shapefiles) and add these fluxes to the groundwater water flow and background nutrient flux. Within each grid cell, GMS multiplied the number of houses and area of fertilized green space with their respective nutrient flux and added these fluxes to the background water flow and nutrient flux stored in the underlying raster gridded maps (e.g., 15x15 m at Hā‘ena and 50x50 m at Ka‘ūpūlehu) [101].

### Linking land and sea models

To link the groundwater and coral reef models and represent the non-point source nature of SGD (Fig 2D), we sub-divided the groundwater model domains into narrow flow tubes (~200 m width) ending in pour points at the shoreline using MODPATH (Fig 3C & 3D) [73,74,102]. The flow tube boundaries were established along groundwater flow path lines and assumed very little exchange of groundwater and dissolved nutrients between them. MODPATH relies on the MODFLOW groundwater flow solution to model particle movement along the simulated track to an endpoint [73,74]. Virtual particles were placed at the pour points along the shoreline and the reverse tracking option was used to delineate groundwater flow paths from the coast to the zones of recharge, through the model domain. The width of the flow tubes captured the spatial distribution of groundwater flow rates and nutrient sources from anthropogenic sources (i.e., land cover/use). The groundwater flow rates varied along coastline features, such as embayments where groundwater flow lines converged, and coastal protrusions where groundwater flow lines dispersed. The smallest land cover type was houses, aggregated into small communities, which formed the smallest spatial unit for nutrient sources. The upland extent of flow tubes was determined by a groundwater elevation contour to be consistent with groundwater flow through aquifers. The length of the flow tubes was based on the boundaries of coastal development. Because coastal development at Hā‘ena was concentrated along the coastal zone, the flow tubes reached 1,000 m inland (Fig 1B). At Ka‘ūpūlehu, the coastal development extended further inland so the length of the flow tubes was 3,500 m (Fig 1C). At the shoreline, the flow tube boundary corresponded to the groundwater model submarine boundary.

Because freshwater (decreases salinity) and nutrients (promote algae growth) affect coral reefs differently [103], we converted the background nutrient concentrations (mg.L^-1^) into nutrient flux (kg.yr^-1^) (i.e., concentration x annual recharge), using the modeled groundwater recharge (m^3^.yr^-1^). This allowed us to model freshwater and nutrients as separate variables and thus quantify the independent effects of freshwater and nutrient discharge on the coral reef indicators. The groundwater discharge and nutrient flux from the grid cells were then computed for each flow tube using the groundwater utility model, ZONEBUDGET [104]. This consisted of adding the water flow and nutrient fluxes from the multiple grid cell values within the boundaries of each flow tube to the groundwater flow (m^3^.yr^-1^) and nutrient flux (kg.yr^-1^) entering the flow tubes from upslope, and discharging those in bulk at each corresponding pour point.

### Modeling terrestrial drivers

We generated terrestrial drivers’ grid maps (60x60 m) by diffusing the modeled groundwater discharge (m^3^.yr^-1^) and nutrient flux (kg.yr^-1^) from each pour point into the coastal zone using ArcGIS (Fig 2E). First, we created a cost-path surface (***c***) to quantify the least accumulative cost-distance (impedance) of moving planimetrically through each cell from each pour point, using a composite of three marine drivers known to affect diffusion (depth [m], distance from shore [m], and wave power [kW.m^-1^]–see ‘Modeling marine divers’ for more details) [26,105]. Then, the spread of groundwater and nutrient values into coastal waters from each pour point was modeled using a decay function (*see* Eq 3), which assigned a portion of the remaining quantity from the previous cell in all adjacent cells, based on the cost-path surface until a maximum distance of 1 km from the shoreline was reached [49,60,106–108]:

$$W_{i}\;=\;L_{p}\times e^{-c^{2}/D_{c}}$$ (3)

where *W* = Grid cell value for diffused groundwater (m^3^.yr^-1^) and nutrients flux (kg.yr^-1^), *L*_*p*_ = Groundwater (m^3^.yr^-1^) and nutrients (kg.yr^-1^) flux at each pour point (obtained from computing the groundwater and nutrient flux by flow tube), *c* = cost-path surface (unitless), *D*_*c*_ = cost-path surface threshold distance from the shore for each decayed groundwater metrics (equivalent to 1,000 m from the shoreline). This approach to modeling SGD is diffusive, and thus, allows for wrap around coastal features, but did not account for nearshore advection that acts to push the SGD in specific directions [49]. We used these diffusive models to derive conservative estimates of SGD plumes, since the nearshore circulation patterns were unknown for our study sites.

We assumed that the nutrient chemistry of the SGD was similar to that of the groundwater. Biogeochemical reactions that could occur, but were not considered in this study are those associated with denitrification and anammox (anaerobic ammonium oxidation). Given that the biogeochemical conversion of N and P to other species requires reducing conditions, the high dissolved oxygen content (dominantly >80%) in the aquifers around the main Hawaiian Islands results in stable oxidized forms of dissolved N and P, which are the dominant species [84,88,91,109]. Thus there is a possibility that we over- and under-estimated the amount of N and P, respectively, particularly at Ka‘ūpūlehu, where wastewater is disposed of through injection wells [88,109]. Due to the very limited coastal water quality data in our model domains (S3 Table), these modeled terrestrial drivers could only be partially ground-truthed at Ka‘ūpūlehu using linear regression (R^2^ and p-value) (Fig 3D). These SGD models were meant to capture general spatial patterns, which could be refined with future SGD measurements.

### Modeling marine drivers

The marine drivers grid maps (60x60 m) were derived from remote sensing and wave model data available for both sites using GIS-based tools (Fig 2F & 2G) [110]. These were identified as important drivers of coral reef benthic and fish communities based on existing literature and local community input (Table 3 & S4 Table). The wave disturbance driver was represented by mean wave power at each site (kW.m^-1^) and derived from a 500 m resolution SWAN hindcast model that spanned 10 years (2000–2009) [111]. Depth and distance from shore were used to account for variation arising from spatial location. Depth was derived from a synthesis of multibeam sonar and LiDAR bathymetry at 5 m resolution, and distance from shore was derived from the statewide coastline map [112,113]. Three types of habitat drivers, representing direct and indirect effects of seafloor topography on benthic and fish communities were also derived from this bathymetry data [113]: (1) habitat topography, (2) habitat complexity, and (3) habitat exposure. Habitat topography metrics, represented by Bathymetric Position Index (BPI) and slope described the position of the reef relative to the surrounding area. These metrics were computed for two neighborhood sizes (60 and 240 m radii) to capture habitat topography at different spatial scales [114,115]. Habitat complexity metrics, represented by rugosity, planar curvature, and profile curvature were computed to describe fine-scale topographic structure. Habitat exposure metrics were used to characterize the direct and indirect effects of water flow due to fine-scale seafloor topography and directionality. These metrics were derived by computing seafloor aspect, the steepest downslope direction of the seafloor measured in degrees. We calculated the circular mean and standard deviation of aspect and converted the circular mean into measures of northness and eastness by calculating the cosine and sine, respectively.

**Table 3:** Description of marine drivers.

Indicator^a^	Driver	Description	Unit
Wave	Power^b^	Mean wave power derived from a 10 year (2000–2009) SWAN hindcast wave model.	kW.m^-1^
Geography	Depth^c^	Mean seafloor depth	m
	Distance to shore^d^	Euclidean distance to the shoreline	m
Habitat topography	BPI^c^	Relative topographic position of a point based its elevation and the mean elevation within a neighborhood (m)	m
	Slope^c^	Maximum rate of change in seafloor depth between each grid cell and its neighbors	Degrees
Habitat complexity	Planar curvature^c^	Seafloor curvature perpendicular to the direction of the maximum slope (mean). Value indicates whether flow will converge or diverge over a point.	Radians.m^-1^
	Profile curvature^c^	Seafloor curvature in the direction of the maximum slope (mean). Value indicates whether flow will accelerate or decelerate over the curve.	Radians.m^-1^
	Rugosity^c^	Measure of small-scale variations of amplitude in the height of a surface (mean). Value range from 1 (flat) to infinity.	Unitless
Habitat exposure	Aspect^c^	Downslope direction of maximum rate of change in seafloor depth between each grid cell and its neighbors (sine circular mean, cosine circular mean, circular standard deviation)	Degrees

Refer to S5 Table for more details on processing methods.

^a^ The marine drivers were generated at 60 m resolution

^b^ SWAN hindcast wave model at 500 m native resolution [113]

^c^ Bathymetry synthesis at 5 m native resolution [113]

^d^ Coastline [112]

### Identifying the drivers differentiating coral reefs

To identify the drivers differentiating the coral reefs between both sites, we used a distance based redundancy analysis (dbRDA) in PRIMER PERMANOVA+ software [116]. The dbRDA routine performed an ordination of the coral reef indicators as a function of the drivers [116,117]. A Euclidean distance similarity measure was used to construct a resemblance matrix of the transformed and normalized benthic and fish indicators. Square root and fourth root transformations were applied to the benthic and fish variables, respectively, to improve normality [63,118]. Environmental drivers were normalized, with highly correlated (r> 0.7) drivers removed from the models. A fitted variation >70% was considered a good fit to the model [116].

### Coral reef modeling

We used BRT to build the coral reef models (Fig 2H) [119]. Tree-based models are effective at modeling nonlinearities, discontinuities (threshold effects), and interactions between variables, which is well suited for the analysis of complex ecological data [120–122]. BRT models can accommodate many types of response variables, including presence/absence, count, diversity, and abundance data [123]. Since the coral reef indicators were all continuous variables, the response variables were modeled using a Gaussian (normal) distribution, and appropriate data transformations (square root for benthic indicators and fourth root for fish biomass) were applied to improve the normality of the response variable distributions. We calibrated the BRT models on coral reef data to determine the most influential drivers (among the simultaneously tested predictors) and estimate the underlying relationship between the modeled indicators and the key drivers using response curves [123,124]. The values of the terrestrial and marine drivers’ grid maps were sampled using bilinear interpolation at the location of each reef survey (start of the transect) in ArcGIS. This approach takes a weighted average of the 4 nearest cell values, thereby accounting for the relative position of the reef surveys on the predictor grids and their different native spatial scales. The values of the coral reef indicators and interpolated terrestrial and marine drivers at these locations were combined in a single data table to calibrate the BRT models.

Each indicator was modeled independently as a function of the terrestrial and marine drivers at each site. First, we calibrated each benthic indicator model as a function of the terrestrial and marine drivers. Then, we calibrated each fish indicator model as a function of the terrestrial and marine drivers and included the empirical abundance of benthic groups as additional predictors in the models for the fish groups. The calibration process used an internal ten-fold cross-validation to maximize the model fit and determine the optimal combinations of four parameters: (1) learning rate (lr); (2) tree complexity (tc); (3) bag fraction (bag); and (4) the maximum number of trees (*see* [123] for more details). We used the percent deviance explained (PDE) and internal ten-fold cross validation PDE (CV PDE) as performance measures of the model optimum. The optimal models explained the most variation in the response variables (i.e., greatest CV PDE) and were selected as the best and final models. The model calibration was conducted in R software using the gbm package [123,125,126]. Spatial autocorrelation of the response variable was tested using Moran’s I Index for both the raw values and the ecological model residuals [127].

### Coral reef predictive mapping

The coral reef predictive maps were generated at 60x60 m to account for the dimensions of the reef survey methods (i.e., 25–50 m transects) and the positional accuracy of global positioning system used to navigate to them in the field [71,72]. Using the calibrated BRT models, we predicted and mapped the distribution of each coral reef indicator on a cell-by-cell basis using the values of the terrestrial and marine drivers at each cell across the coral reef model domains. The boundaries of the coral reef model domains comprised the lateral boundaries of the *ahupua‘a* to capture the spatial extent of this management unit and the offshore boundary corresponded to the maximum surveyed depth (i.e., 15 m at Hā‘ena and 22 m at Ka‘ūpūlehu) (Fig 3C & 3D). This spatial predictive modeling method is static in nature, so we did not account for exchange between grid-cells, such as fish movement. First, we spatially predicted each benthic indicator as a function of their key drivers. Then, we spatially predicted the fish indicators as a function of their key drivers, including the predicted distribution of the benthic indicators. The predicted values of the benthic and fish grid maps were sampled using bilinear interpolation at the location of each reef survey (start of the transect) in ArcGIS, thereby accounting for the relative position of the reef surveys on the predicted grids. The values of the interpolated predictions and surveyed coral reef indicators at these locations were compared with a linear regression (R^2^ and p-value). Then, we overlaid the predicted maps with the survey points values for each indicator using the same color ramp scale for the legend to enable visual comparison. The spatial predictions were performed in the R software using the dismo and raster packages [126,128,129].

### Identifying priority areas for management

Once calibrated for each site, we used this framework to identify priority areas on land where management actions that reduce or limit additional nutrient inputs can promote coral reef resilience in the face of climate change. We considered a suite of criteria derived from the drivers, coral reef models, and coral reef indicators specific to each site. Based on the assumption that corals in shallow areas are more vulnerable to bleaching from increases in sea surface temperatures [10], we focused on shallow depths at both sites (<5 m). We further considered areas with above-average (based on individual site means) coral, macroalgae or CCA, and turf algae cover, as well as high nutrient inputs and low wave mixing (based on the high and low tercile, respectively), assuming these criteria would also contribute to vulnerability to phase shifts resulting from climate induced bleaching [10,16,17,26]. Because P was not shown to be a driver of the benthic community at Hāʻena, we did not consider it here. Likewise, we did not consider wave power for Kaʻūpūlehu. For Hāʻena, we classified areas with percent cover of coral > 11.4%, macroalgae > 9.4%, and turf algae >50.0%, and areas subject to N >1,322.7 kg.yr^-1^ and wave power <19,860 kW.m^-1^. At Kaʻūpūlehu, we classified areas with percent cover of coral > 18.4%, CCA > 4.1%, and turf algae >44.4%, and areas exposed to N >2,940.8 kg.yr^-1^ and P >325.2 kg.yr^-1^. Using raster calculations in ArcGIS, we identified coral reef areas where these criteria overlapped and were, therefore, most susceptible to future coral bleaching and nutrient impacts. By matching these coral reef areas with corresponding flow tubes with high nutrients derived from anthropogenic activities, we located priority areas on land where nutrient inputs should be limited or reduced.

## Results

### Groundwater models

**Fig 4:**
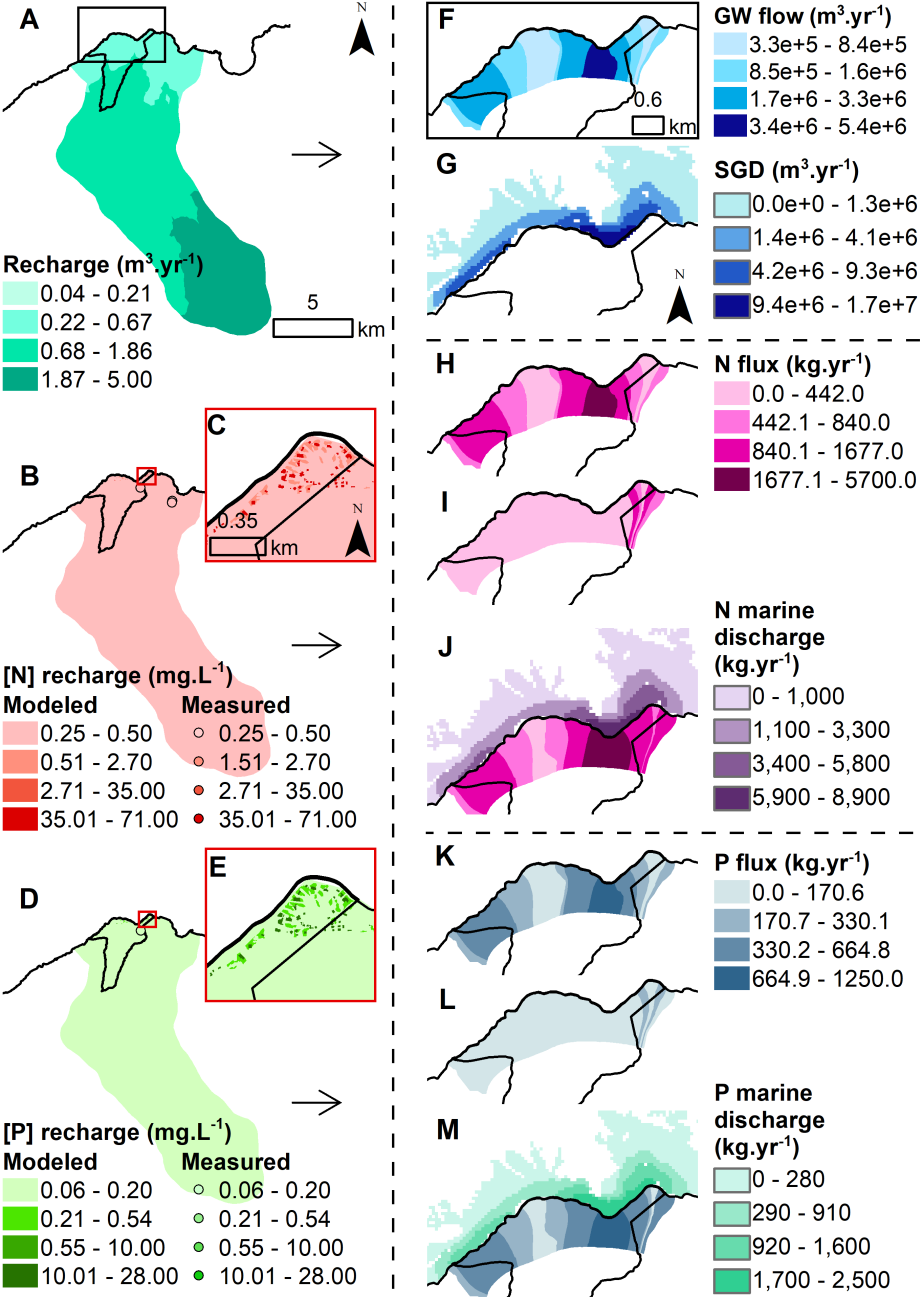
Groundwater recharge, associated nutrient concentrations, and groundwater discharge flux (i.e. terrestrial drivers) at Hā‘ena. (A) Groundwater recharge (m^3^.yr^-1^). (B) Groundwater recharge N concentration (mg.L^-1^) (with enlarged inset C). (D) Groundwater recharge P concentration (mg.L^-1^) (with enlarged inset E). The modeled recharge (B) N and (D) P nutrient concentrations maps are overlaid with the GW survey points using the same color ramp for visual comparison. (F) Modeled coastal groundwater flow (m^3^.yr^-1^) coupled with (G) the SGD (m^3^.yr^-1^). (H) Background, (I) human-derived, and (J) total N flux (kg.yr^-1^) by flow tube combined with N marine discharge plume (kg.yr^-1^). (K) Background, (L) human-derived, and (M) total P flux (kg.yr^-1^) by flow tube combined with P marine discharge plume (kg.yr^-1^)The R^2^ and p-value compare the measured N and P concentrations (mg.L^-1^) in coastal waters and modeled (J) N and (M) P marine discharge (kg.yr^-1^) (see S1 Fig for linear regressions).

**Fig 5:**
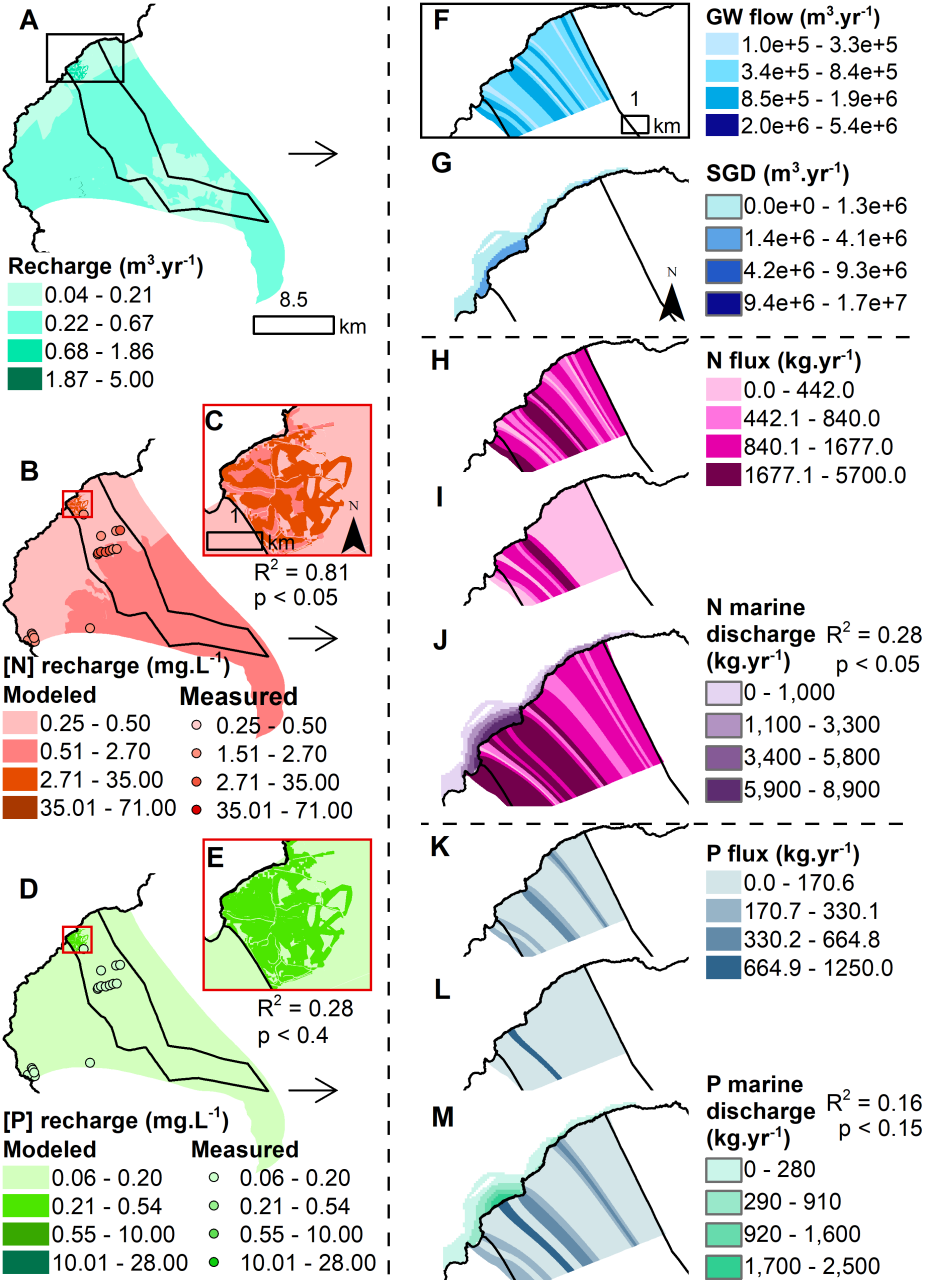
Groundwater recharge, associated nutrient concentrations, and groundwater discharge flux (i.e. terrestrial drivers) at Ka‘ūpūlehu. (A) Groundwater recharge (m^3^.yr^-1^). (B) Groundwater recharge N concentration (mg.L^-1^) (with enlarged inset C). (D) Groundwater recharge P concentration (mg.L^-1^) (with enlarged inset E). The modeled recharge (B) N and (D) P nutrient concentrations maps are overlaid with the GW survey points using the same color ramp for visual comparison. The R^2^ and p-value compare the measured and modeled (E) N and (I) P concentrations at Ka‘ūpūlehu (see S1 Fig for linear regressions). (F) Modeled coastal groundwater flow (m^3^.yr^-1^) coupled with (G) the SGD (m^3^.yr^-1^). (H) Background, (I) human-derived, and (J) total N flux (kg.yr^-1^) by flow tube combined with N marine discharge plume (kg.yr^-1^). (K) Background, (L) human-derived, and (M) total P flux (kg.yr^-1^) by flow tube combined with P marine discharge plume (kg.yr^-1^)The R^2^ and p-value compare the measured N and P concentrations (mg.L^-1^) in coastal waters and modeled (J) N and (M) P marine discharge (kg.yr^-1^) (see S1 Fig for linear regressions).

The groundwater model results showed differences in recharge rates and nutrient concentrations between both sites. Groundwater recharge was much higher at Hā‘ena (ranging from 0.11 to 4.97 m.yr^-1^) compared to Kaʻūpūlehu (ranging from 0.04 to 0.69 m.yr-1) (Figs 4A & 5A). The background N concentrations were higher at Kaʻūpūlehu (0.25–2.70 mg.L^-1^), compared to Hā‘ena (0.5–0.85 mg.L^-1^) (Figs 4B & 5B). While the background P concentrations were similar for Hā‘ena (0.09–0.20 mg.L^-1^) and Kaʻūpūlehu (0.10–0.20 mg.L^-1^) (Figs 4D & 5D). The key sources of human-derived nutrients were wastewater from houses on cesspools at Hā‘ena (Fig 4C & 4E) and the golf course and wastewater from the injection well at Kaʻūpūlehu (Fig 5C & 5E). The comparison of measured and modeled nutrient concentrations at Ka‘ūpūlehu, indicated that the N model performed better compared to the P model (Fig 5B & 5D, S1 Fig). Data was insufficient to allow for a similar comparison at Hā‘ena.

### Terrestrial and marine coral reef drivers

**Fig 6:**
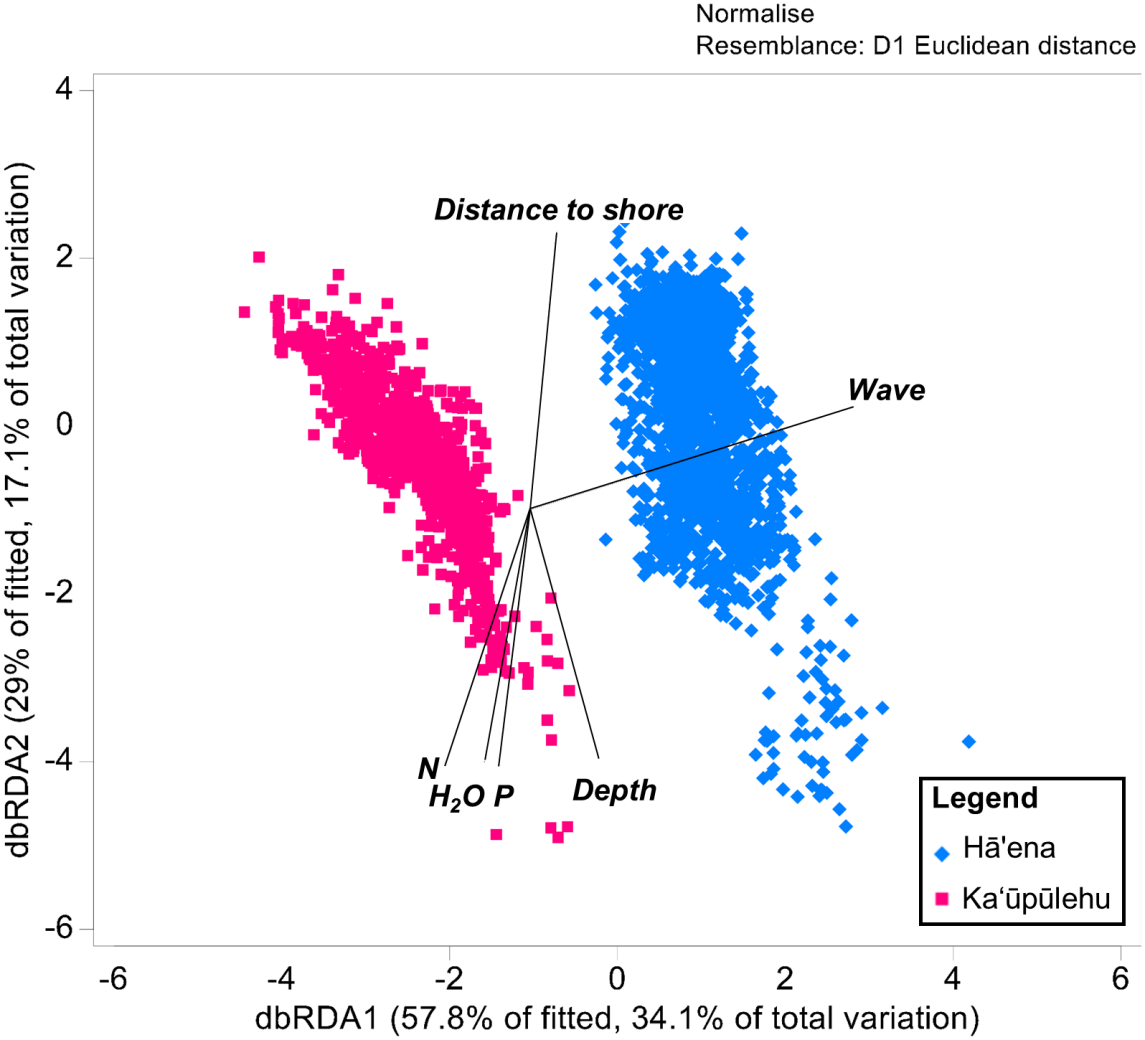
dbRDA of the coral reef communities. Ordination plot illustrating the relationship between terrestrial and marine drivers that best explain the variation of benthic and fish indicators in Hā‘ena and Kaʻūpūlehu. The dbRDA vectors show the drivers explaining a significant proportion of the variation. The drivers differentiating the coral reef communities at Hā‘ena from Ka‘ūpūlehu are: wave power, distance to shore, depth, groundwater (H_2_O) and nutrients (N and P).

**Fig 7:**
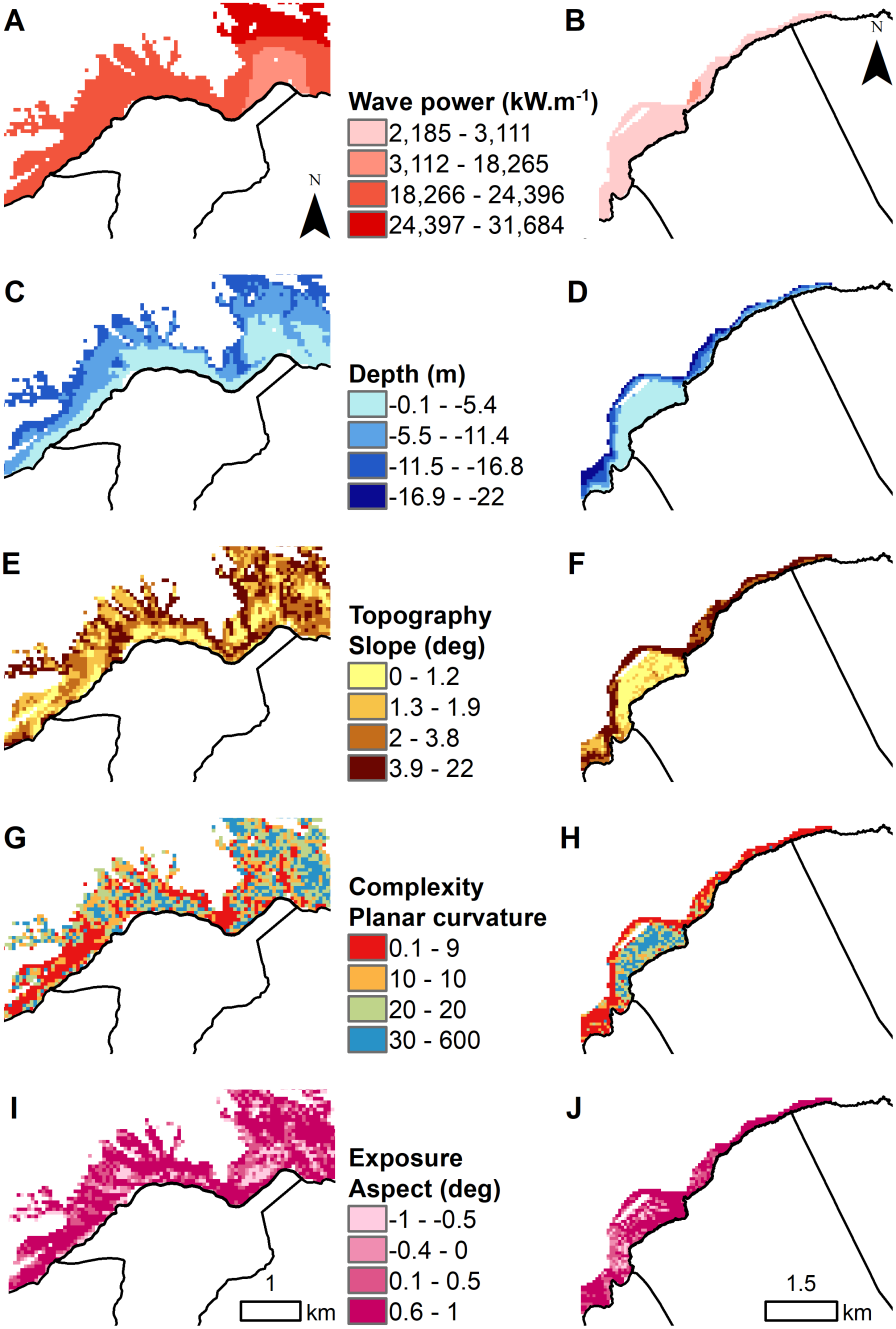
Marine drivers of coral reefs at Hā‘ena and Ka‘ūpūlehu. The marine drivers are represented by (A-B) wave power (kW.m^-1^), (C-D) depth (m), (E-F) habitat topography (slope [degree]), (G-H) habitat complexity (planar curvature), and (I-J) habitat exposure (aspect [degree]) at Hā‘ena and Ka‘ūpūlehu, respectively.

According to the dbRDA (Fig 6), Hā‘ena and Ka‘ūpūlehu coral reefs were well separated in ordination space based on terrestrial (freshwater, N, and P) and marine (wave, distance from shore, and depth) drivers (presented in Figs 4, 5 & 7). The first axis accounted for 57.8% of the fitted variation (corresponding to 34.1% of the total variation) and the second axis accounted for 29% of the fitted variation (equivalent to 17.1% of the total variation). The first axis was primarily correlated with wave power, thereby separating the coral reefs exposed to high wave power at Hā‘ena ($\bar{X}=21,697\pm 4,119$), from the coral reefs sheltered from wave power at Ka‘ūpūlehu ($\bar{X}=2,756\pm 186$) (Fig 7A & 7B). The second axis was positively correlated with distance from shore and negatively correlated with depth, thereby separating the wider and shallower eroded island shelf of Hāʻena (distance to shore $\bar{X}=594.5\pm 422.8$ and depth $\bar{X}=-7.7\pm 6$) from the narrow and steep island shelf of Ka‘ūpūlehu (distance to shore $\bar{X}=269.5\pm 187.1$ and depth $\bar{X}=-8\pm 4.7$) (Fig 7C & 7D). While not identified as primary marine drivers differentiating the sites, habitat topography metrics indicated that the reef slope was steeper at Kaʻūpūlehu (slope_60_ $\bar{X}=3.4\pm 2.4$) compared to Hā‘ena (slope_60_ $\bar{X}=2.8\pm 1.8$) (Fig 7E & 7F), while habitat complexity was higher at Hā‘ena (planar curvature $\bar{X}=18.1\pm 9.3$) compared to Kaʻūpūlehu (planar curvature $\bar{X}=13.8\pm 9.9$) (Fig 7G & 7H). In terms of habitat exposure, Hā‘ena (Aspect $\bar{X}=0.4\pm 0.6$) was more exposed than Kaʻūpūlehu (Aspect $\bar{X}=0.6\pm 0.5$) (Fig 7I & 7J).

The second axis was also negatively correlated with the terrestrial drivers. It indicated that groundwater discharge (represented by freshwater) was higher at Hā‘ena (57.1 million m^3^.yr^-1^ or 10,279 m^3^.m^-1^.yr^-1^) than Kaʻūpūlehu (22.7 million m^3^.yr^-1^ or 3,085 m^3^.m^-1^.yr^-1^), with higher input in bays at both sites (Figs 4F & 5F). Likewise, P flux was higher at Hā‘ena (13,050 kg.yr^-1^ or 2.2 kg.yr^-1^.m^-1^), compared to Ka‘ūpūlehu (6,760 kg.yr^-1^ or 0.8 kg.yr^-1^.m^-1^) (Figs 4M & 5M). Conversely, N flux was higher at Ka‘ūpūlehu (55,540 kg.yr^-1^ or 7.1 kg.yr^-1^.m^-1^), in comparison to Hā‘ena (36,320 kg.yr^-1^ or 6.0 kg.yr^-1^.m^-1^) (Figs 4G & 5G). The fraction of human-derived nutrient flux delivered to the coast was lower at Hā‘ena (N = 16.4% and P = 10.7%) than Ka‘ūpūlehu (N = 31.7% and P = 34.9%) (Figs 4I, 4L, 5I & 5L). In contrast, the fraction of natural-derived nutrient flux delivered to the coast was higher at Hā‘ena (N = 83.6% and P = 89.3%) than Ka‘ūpūlehu (N = 68.3% and P = 65.1%) (Figs 4H, 4K, 5H & 5K).

### Coral reef models

**Fig 8:**
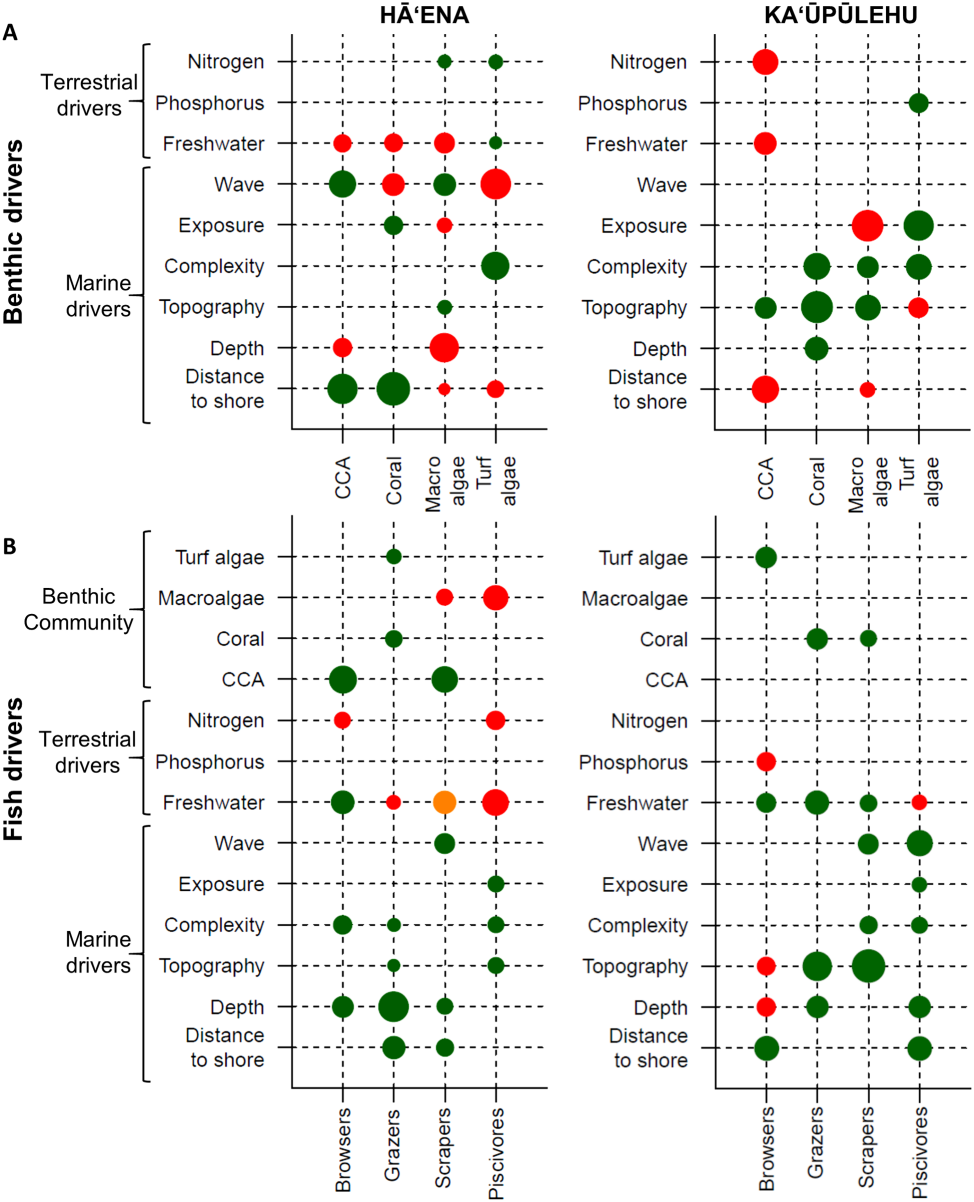
Coral reef predictive models. (A) Benthic and (B) fish predictive models for each site. The benthic and fish indicators are represented along the X axes. The terrestrial and marine drivers, and benthic community (for the fish models) are represented on the Y axes. The marine drivers include metrics related to wave power, habitat exposure, complexity, topography, and local geography (depth and distance to shore). The bubble size represents the relative percent contribution of each driver and the color indicates whether the relationship between the indicator and the driver is positive (green), concave/convex (yellow), or negative (red). Refer to S2–S7 Figs for more details on these relationships.

The calibration and cross-validation of coral reef BRT models for Hā‘ena explained 34–74% of the PDE and 10–51% of the CV PDE, respectively (S6 Table). At Ka‘ūpūlehu, the calibration and cross-validation of coral reef models explained 21–60% of the PDE and 5–26% of the CV PDE, respectively. Analysis of the residuals from the final coral reef models showed no spatial autocorrelation (Moran’s I Index p > 0.1). In terms of the terrestrial drivers, the coral reef models identified that groundwater discharge (represented by freshwater) was a key driver of coral reefs at Hā‘ena, while nutrients played a more important role for coral reefs at Ka‘ūpūlehu (Fig 8). At Hā‘ena, freshwater had a negative effect on CCA, coral, and macroalgae, but was positively related to turf algae. Turf and macroalgae were weakly, yet positively related to N. Conversely, N had a negative effect on browser and piscivore biomass. At Ka‘ūpūlehu, N had a negative effect on CCA, while P had a positive effect on turf algae and a negative effect on browsers. The effects of freshwater varied across fish indicators, as well as between sites.

In terms of the marine drivers, the coral reef models identified wave power, depth, and distance to shore as key drivers of coral reefs at Hā‘ena (Fig 8). Wave power had a positive effect on CCA, macroalgae, and scrapers, but a negative effect on coral and turf algae. Coral was, however, positively associated with habitat exposure. CCA, coral, and most herbivores were positively associated with distance from shore, whereas turf and macroalgae showed a negative relationship. Depth had a negative effect on CCA and macroalgae. The fish indicators were more strongly associated with CCA, but scrapers were also negatively associated with macroalgae, while grazers positively associated with turf algae and coral. At Ka‘ūpūlehu, the coral reef models identified habitat topography and complexity, as well as depth and distance to shore as key drivers of coral reefs (Fig 8). Although wave power had a positive effect on scraper and piscivore biomass, it had no effect on the benthic indicators. CCA and macroalgae were negatively related to distance to shore, whereas browsers and piscivores showed a positive relationship. The fish indicators were more strongly associated with coral and turf algae. Most benthic and fish indicators were positively associated with steeper, deeper reef slopes and more complex habitat, except for turf algae and browsers, which were negatively associated with habitat topography.

### Coral reef predictive maps

**Fig 9:**
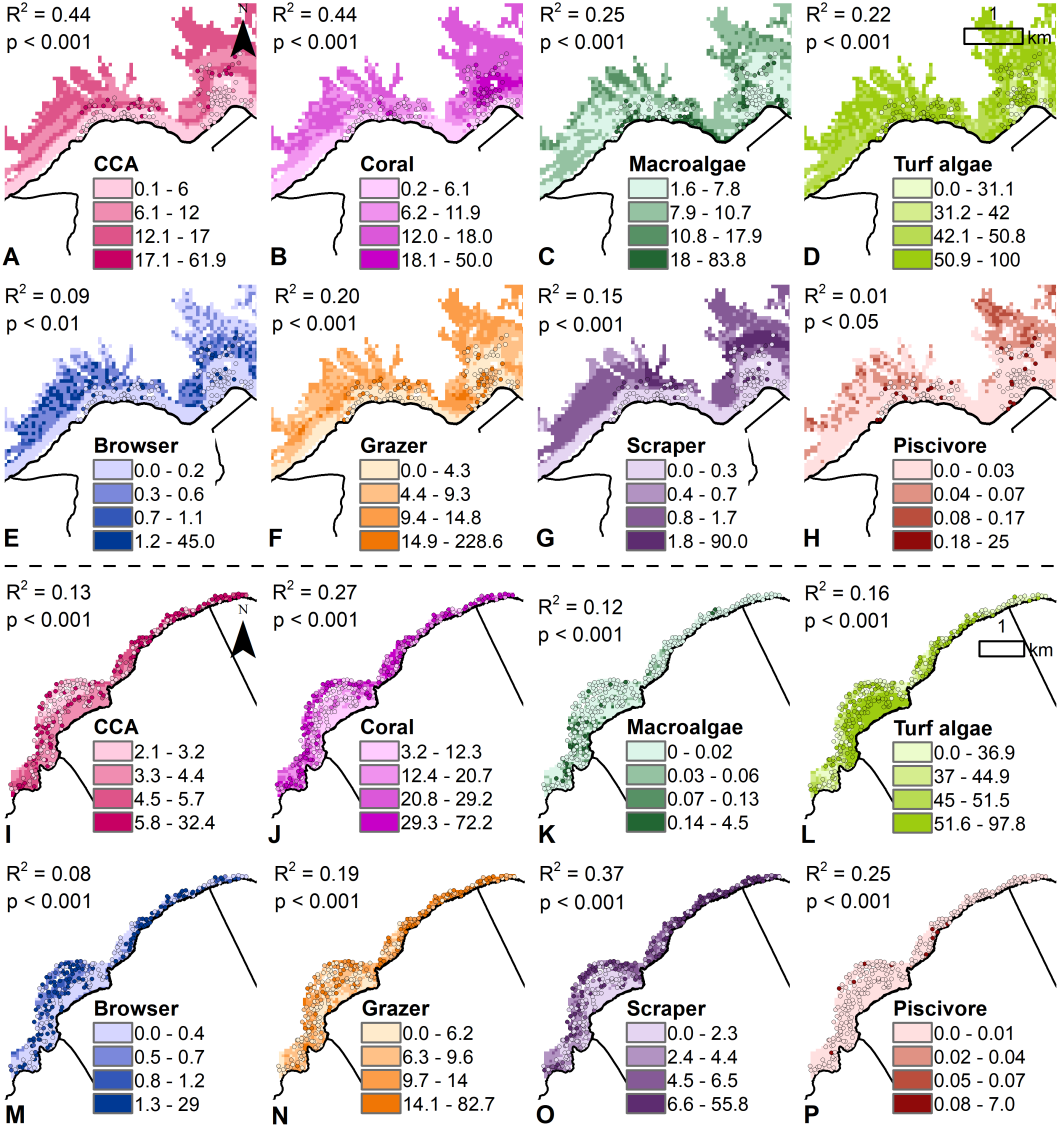
Observed and predicted distribution of coral reef indicators at Hā‘ena and Ka‘ūpūlehu. Benthic indicators at (A-D) Hā‘ena and (I-L) Ka‘ūpūlehu are measured in % cover and the fish indicators at (E-H) Hā‘ena and (M-P) Ka‘ūpūlehu are measured in g.m^-2^. The predicted maps are overlaid with the survey points using the same color ramp for visual comparison, combined with the R^2^ and p-values.

Based on the key drivers and their relationship with the coral reef indicators at Hā‘ena (i.e., freshwater, wave power, depth, and distance to shore) (Fig 8), the coral reef models predicted a benthic community with high CCA cover, particularly along the wave-exposed fore-reefs away from freshwater influence; higher coral cover was restricted to the sheltered back-reef areas; higher macroalgae was concentrated in the nearshore areas, close to sources of nutrients; while turf algae was high and more widespread (Fig 9). The coral reef models predicted a fish community with many grazers but few browsers, scrapers, and piscivores, with higher biomass for all indicators in more complex habitat, deeper waters, and away from the shore (Fig 9). Based on the comparison with the empirical surveys with the spatial predictions, all the indicators, except for the piscivores, showed a statistically significant relationship (Fig 9). The R^2^ was higher for CCA and coral predictions compared to the turf and macroalgae predictions, and the grazers and scrapers predictions performed better than the browsers and piscivores. Those trends were consistent with their relative empirical abundance and biomass at the survey sites (Fig 9).

Based on the key drivers and their relationship with the coral reef indicators at Ka‘ūpūlehu (i.e., nutrients, habitat topography and complexity, depth, and distance to shore) (Fig 8), the coral reef models predicted a benthic community with low CCA cover, restricted to the nearshore areas away from nutrient discharge; high coral cover, particularly along the reef slopes; low macroalgae cover restricted to the nearshore areas; and abundant turf algae cover on the reef flat, near nutrient discharge (Fig 9). The coral reef models predicted a fish community with many grazers and scrapers but few browsers and piscivores, with higher biomass for all indicators in more complex habitat, deeper waters, and away from the shore (Fig 9). Based on the comparison of the empirical surveys, the spatial predictions of all the indicators showed a statistically significant relationship (Fig 9). The R^2^ was higher for coral and turf algae predictions compared to CCA and macroalgae predictions, and the scrapers and piscivore predictions performed better than the browsers and grazers. Those trends were also consistent with their relative empirical abundance and biomass at the survey sites (Fig 9).

### Priority areas for management

**Fig 10:**
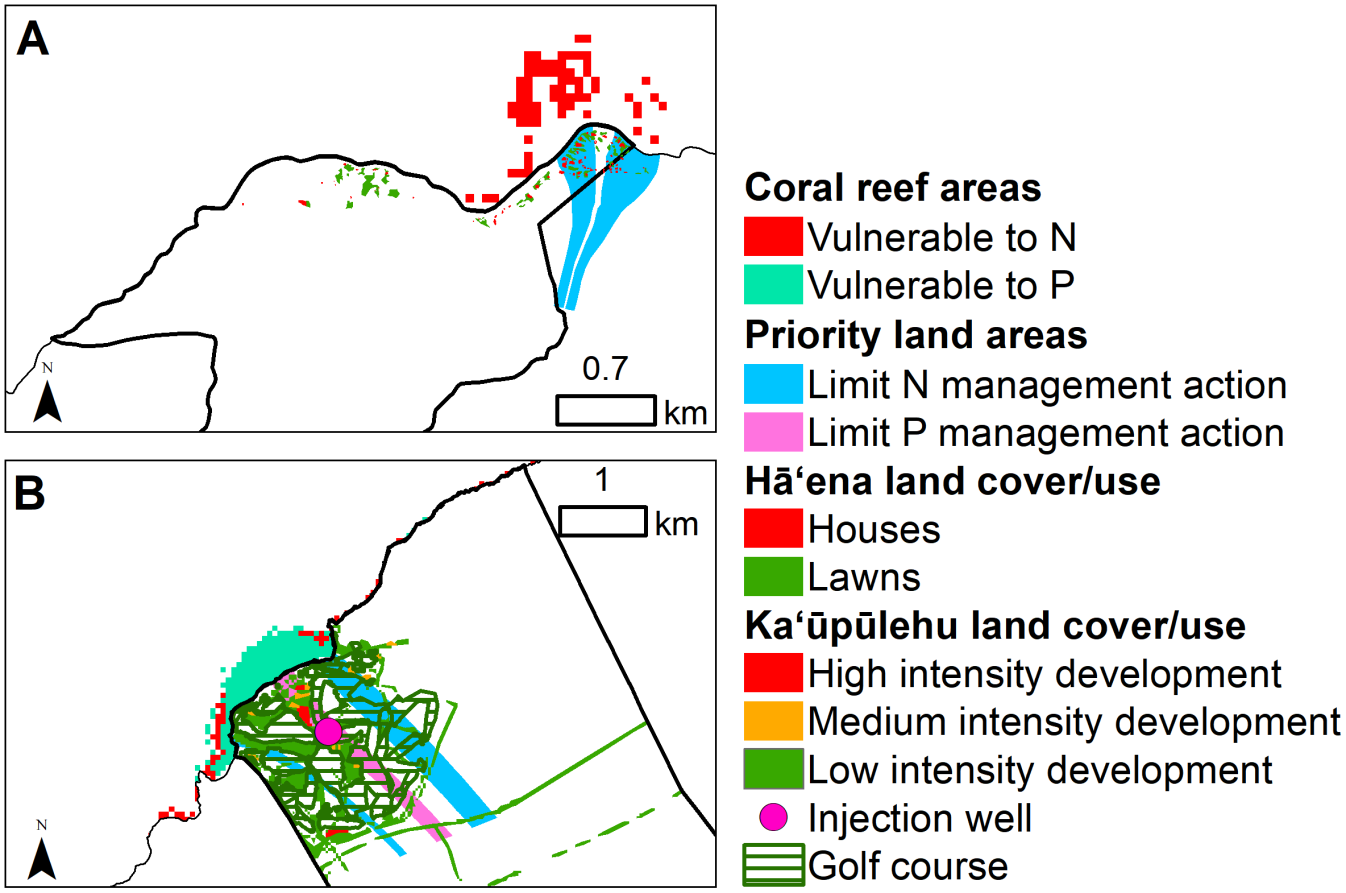
Coral reef areas vulnerable to land-based nutrients and priority land areas at Hā‘ena and Ka‘ūpūlehu. (A) Hā‘ena and (B) Ka‘ūpūlehu coral reef areas vulnerable to nutrients (N and P) combined with the priority land areas with the highest human derived nutrients and therefore, where management action should limit N and/or P inputs.

By combining the selected criteria at Hāʻena, we found that the back-reef of Makua was vulnerable to nutrient inputs due to high exposure to N, limited wave mixing, and abundant benthic algae, as well coral bleaching due to high coral cover and shallow depth (Fig 10A). On land, the flow tubes located to the east of the site deliver the highest N flux. Based on the location of vulnerable coral reef areas, the cesspools located within these flow tubes should be prioritized for upgrade to septic systems. At Kaʻūpūlehu, we found that the coral reef areas more vulnerable to N input were located around the edges of existing development and along the reef slopes north of the development (Fig 10B). We also found that the reef flat located downstream from the development is more vulnerable to P input. On land, the flow tubes located to the north and south ends of the development, discharged the highest N flux in the small bays there, while the flow tubes located below the injection well discharged the highest P flux.

## Discussion

To support ridge-to-reef management in high oceanic islands, this study developed a linked land-sea modeling framework that connects land cover/use to coral reefs through groundwater enriched nutrients at fine spatial resolution. We applied this framework in two *ahupua‘a* subject to different natural disturbance regimes to compare and contrast the effects of terrestrial and marine drivers on coral reefs. Our results indicate that the terrestrial and marine drivers differed between sites due to their natural disturbance regimes and different island age. Hā‘ena is primarily influenced by large-scale drivers (high rainfall and wave power), while Ka‘ūpūlehu is mostly governed by local drivers (habitat and nutrients). Consistent with previous studies [22,130–132], our coral reef models showed that the high disturbance regime of Hā‘ena has shaped a coral reef community dominated by CCA with cover of high turf algae and many grazers, while the low wave disturbance regime of Ka‘ūpūlehu has allowed for the accretion of a coral dominated community with high turf and many grazers and scrapers. Similar to other studies [16,17,26,133–135], our coral reef predictive models showed that land-based nutrients can increase benthic algae, inhibit reef calcifiers (CCA), and decrease the biomass of locally important fishes. This study shows how coral reefs can differ under the influence of different natural disturbance regimes combined with local-scale terrestrial and marine drivers, thereby reinforcing the need for place-based ridge-to-reef management.

### Groundwater as the land-sea linkage

Our groundwater flow modeling results reflected the rainfall patterns from each site, where recharge at Hā‘ena was much higher than at Ka‘ūpūlehu, and resulted in larger SGD in embayments [37,136]. Given that nutrient concentrations in groundwater depend on rates of recharge, the higher rates of recharge at Hā‘ena resulted in more dilution and lower N concentrations, compared to Ka‘ūpūlehu [137]. Our coastal nutrient flux discharge modeling results confirmed that groundwater at Ka‘ūpūlehu has high natural N flux, despite the existing land cover consisting only of barren rock, shrubland, and native and invasive forests [138]. Some have hypothesized that the groundwater may be geothermally altered, but the exact source of N remains unknown [78,138]. Ka‘ūpūlehu results were consistent with other areas on the dry leeward side of Hawaiʻi Island, where coastal groundwater nutrient fluxes are high (e.g., N:2,000 and P: 200 kg.ha^-1^.yr^-1^), compared to other less dry high latitude oceanic islands, such as South Korea (N:1,100 and P:20 kg.ha^-1^.yr^-1^) [59,139]. Hā‘ena nutrient flux modeling results were consistent with other wet and rural *ahupua‘a* along the Main Hawaiian Islands chain, such as Hanalei on the windward side of Kaua‘i, Kahana on the windward side of O‘ahu, and the south shore of Moloka‘i [59,62,107]. At Hā‘ena, a large fraction of the total nutrient flux is derived from natural processes due to abundant rainfall. Because we assumed constant background nutrient concentrations, the higher groundwater discharge rates resulted in higher background nutrient flux. Similar to other studies [60,61,140], our results indicated that SGD on oceanic islands can be a primary vector for land-based nutrients to coral reefs.

### Anthropogenic sources of nutrients

This study showed that wastewater disposal via cesspools at Hā‘ena was the major source of human-derived nutrients. The impact of sewage discharge on coral reefs has been recognized as a major environmental problem in Hawaiʻi [103,141], as well as in regions such as Reunion and Mauritius [122], the Red Sea [142], Florida Keys [143], and the Great Barrier Reef [144]. While, revised wastewater regulations declared a statewide ban of new cesspools in Hawai‘i in 2016 (HAR Title 11, Chapter 62), they currently represent the most prevalent wastewater disposal system across the main Hawaiian Islands (e.g., 76% and 84% of the OSDS currently used on Kaua‘i and Hawai‘i Island, respectively) [145], and have been recognized as a primary driver of groundwater and nearshore water quality degradation [146]. At Ka‘ūpūlehu, we identified the golf course and injection well as the major sources of nutrients. Studies elsewhere in Hawai‘i have also shown that nutrient concentrations can be significantly higher in proximity to golf courses [60,106,147].

### Effect of SGD on coral reefs

Freshwater input from SGD can reduce salinity in shallow waters [15,60,89]. Higher freshwater input at Hā‘ena played an important role in structuring coral reefs. Consistent with their ecology and salinity tolerance [15,148,149], CCA and coral cover were lower near high freshwater inputs. Conversely, decreases in salinity have been shown to directly promote turf algae growth or indirectly hinder competition for space by other species [150]. Freshwater input had a mixed effect on the distribution of the fish indicators, which may be due to the fact that fishes are mobile and tolerate a wider range of salinity, which varies among species [151]. The ecological responses of turf, and macroalgae to nutrients suggested that Hā‘ena may be N-limited, as was shown in nearby Hanalei Bay [106], while Ka‘ūpūlehu may be P-limited, as was found in Honokōhau Bay, also located on the leeward side of Hawai‘i Island [58]. Vermeij et al. [134] showed that local nutrient enrichment can foster turf algae overgrowth and reduce CCA and coral recovery after disturbances, through loss of space availability [152]. Similarly, our results showed that macroalgae, but especially turf algae, may have a competitive advantage over corals and CCA under a future scenario of land-based nutrient increase in nearshore waters, particularly at Ka‘ūpūlehu and the back-reefs of Hā‘ena. Naturally more dominant and competitive under the higher wave disturbance regime in the central Pacific region [22,67,132], turf algae can proliferate rapidly and lead to phase shifts when exposed to land-based nutrients [16,17,26]. Consistent with their ecological role, grazers at Hā‘ena and browsers at Ka‘ūpūlehu appear to be controlling turf algae, demonstrating their importance for coral reef resilience [16,17,20].

### Effect of wave power on coral reefs

On Hawaiian reefs, wave power is a key driver controlling coral growth, reef development, and the structure of coral reef communities [22,131,153]. CCA has been found to be more dominant and competitive under high wave disturbance regimes on coral reefs in the central Pacific region [22,67,132]. The coral and CCA abundance patterns at both sites indicated that CCA may be out-competed by coral under low wave conditions suited to coral growth, but flourish in high wave conditions adverse to coral growth [130,154,155]. Exposed to high wave power, the benthic community on the fore-reefs at Hā‘ena is dominated by CCA, while coral growth is primarily restricted to the sheltered back-reef of Makua. By contrast, coral growth at Ka‘ūpūlehu was more widespread across the reef slope. The effect of wave disturbance on fish populations is not well studied, due to the challenges of conducting field work in high wave environments [156–158]. Our results suggest that fishes may benefit from reduced access due to localized wave action at both sites, implying that wave power provides protection from fishing pressure [156,159,160]. These patterns have been observed elsewhere across the Hawaiian Archipelago, where coral reefs in wave exposed settings are often suppressed to a thin veneer and support high fish biomass, while coral reefs in sheltered areas have accreted slowly over time and support lower fish biomass [22,159,161].

### Effect of habitat on coral reefs

Owing to their island age coupled with their natural disturbance regimes, coral reef habitats at Hā‘ena and Ka‘ūpūlehu exhibit different topographies and complexities. Coral reefs on young islands form relatively narrow fringes, such as in Ka‘ūpūlehu, while coral reefs around older islands, form wider and shallower reef flats, such as in Hā‘ena [153]. Many studies have shown that habitat topography and complexity are primary drivers controlling coral reefs [130,158,159,162], as shown in Ka‘ūpūlehu, compared to Hā‘ena where on habitat topography and complexity were less important. At Ka‘ūpūlehu, CCA, coral cover, and fish biomass were generally high along the reef slopes, while turf and macroalgae cover was higher on the reef flats. Our results show that local-scale habitat characteristics played an important role in shaping these coral reefs, which was emphasized in the low natural disturbance regime at Ka‘ūpūlehu.

### Management implications

At first glance, Ka‘ūpūlehu is more susceptible to nutrient inputs from coastal development and coral bleaching due to high levels of background N in groundwater, combined with limited dilution and mixing from low rainfall and wave power, and high coral and turf algae cover. Based on the location of the vulnerable coral reef areas, our results suggest monitoring the effect of N discharge from the flow tubes located upslope from Uluweuweu and Kahuwai bays and P discharge from the central flow tube beneath the injection well. Currently, Hā‘ena is rural with limited development or agriculture, therefore most of the nutrient discharge comes from natural processes, with the exception of land areas to the east of the *ahupua‘a* where nutrient discharge is largely human-derived. Although Hā‘ena benefits from mixing and dilution due to high freshwater and wave power, the back-reef areas are shallow, sheltered from waves, exposed to natural and human-derived nutrients, and support high coral and algae cover. For these reasons, the back-reef areas of Hā‘ena are expected to be more vulnerable to coral bleaching and algae overgrowth due to nutrient inputs from existing and future coastal development.

The communities in both places have initiated the protection of herbivores, through marine closures, which can offset some of the effects of nutrients by controlling algae cover [16,17,163]. However, to ensure coral reef resilience in a changing climate, land-based nutrients inputs should also be addressed. Using this framework to inform resilience management through a ridge-to-reef approach, we identified priority areas on land where limiting nutrient inputs could prevent increase in benthic algae and promote chances of coral recovery post-bleaching impacts. At Hā‘ena, management actions could focus on upgrading cesspools located upstream from Makua back-reef, which has been shown to be a nursery habitat or *Pu‘uhonua* for fishes [71], and is protected as such under the management plan of the Community Based Subsistence Fisheries Management Area [164]. At Ka‘ūpūlehu, management actions could focus on minimizing increase in P from the injection well discharge and that best management practices are employed for fertilizer application on green spaces located upstream from Uluweuweu bay and Kahuwai bay, which was identified by the Ka‘ūpūlehu community to contain a groundwater spring (*Wai a Kāne*) of cultural and historical importance [165].

### Limitations and future research

Given that this framework was calibrated on existing data, some models could not be validated, or only partially validated, due to limited or lack of data. At Hā‘ena, the groundwater model was parametrized with limited groundwater samples and the coastal discharge models could not be validated due to lack of coastal water quality data. At Ka‘ūpūlehu, the coastal discharge models were partially ground-truthed due to limited coastal water quality data. In addition, we used existing wave data to represent mixing effect from wave action on the diffusion of the SGD, given circulation data was not available for our study sites. To strengthen this method, future work using this approach should include groundwater sampling with SGD measurements, incorporate nearshore circulation information, and couple coral reef surveying with water quality sampling. Thus more refined groundwater models and reliable maps of coastal water quality could be generated. Additionally, our predictive coral reef models were calibrated on contemporary data sets and the derived relationships (response curves) should be further compared against historical data trends for validation. Although this framework was developed based on limited water quality data, we had access to comprehensive datasets for the coral reef models. This allowed us to ground-truth the predicted maps of our resilience indicators, which were the final output of the modeling framework.

Species composition and relative abundance can affect the predictability of selected indicators [166], as illustrated by the differences in observed abundance of CCA and coral between with our study sites. However, the same coral reef survey methods were used to record benthic and fish data at both sites, thus eliminating a potential source of bias. To improve the predictions of the coral reef models, future research in those locations should couple coral reef surveys with water quality and oceanographic conditions (e.g., waves or currents). In light of these caveats, these priority areas should be seen as target zones for wastewater management and further investigation of land-sea impacts. Because these models were developed at high spatial resolution in places where communities are stewards of the environment, we leveraged input from local community members and their observations to further ground-truth our maps and priority areas. The fact that the areas identified as vulnerable coincided with local observations from community members provided additional confidence to our recommendations.

## Conclusions

Managers need spatially-explicit place-based models to better understand the impact of anthropogenic drivers on coral reefs and manage them more effectively. Empirical data provide point data at the location of the survey, but do not provide a continuous surface to support spatial prioritization of management actions [167]. Tools that provide visualization and quantify potential impacts are needed to better manage coral reefs [11,168]. The linked land-sea modeling framework presented here can help managers evaluate the spatial variation and influence of terrestrial and marine drivers, mediated by anthropogenic activities, on coral reefs, and prioritize management actions accordingly. Although these linked land-sea models were built to understand the land-sea linkages specific to these places, many of the processes, ecological effects, and management actions, we described can be generalized to other oceanic island environments comprised within this spectrum of natural disturbance regimes. Additionally, when calibrated for a place and assuming the fundamental ecological relationships are constant over time, this framework can be used to forecast and assess indicator distributions based on land cover/use change, marine closures, and climate change scenarios.

## Supporting information

S1 File Copyright permission Charles Fletcher. (PDF)

S1 Table Modeling framework response variables description. Benthic (% cover) and fish biomass (g.m^-1^) coral reef indicators were derived from the coral reef surveys and used as response variables in the coral reef models.
(DOCX)

S2 Table Fish species composition per functional groups. (DOCX)

S3 Table Coastal water quality data at Ka‘ūpūlehu. See Carlson and Wiegner [169] for more details on sample collection, processing, and analytical methods.
(DOCX)

S4 Table Response variables and drivers’ relationships. This table provides the hypothesized relationships between the drivers and coral reef indicators.
(DOCX)

S5 Table Modeling framework predictor variables description and processing methods. This table provides a description of all the predictor variables modeled in the coral reef models. Each metric is classified by type (terrestrial drivers or marine drivers) and assigned a code for modeling. The table below indicates the data source and analytical tool used to generate each metric. Refer to Stamoulis & Delevaux et al. [110] for more details on processing methods.
(DOCX)

S6 Table Coral reef predictive model performance per indicator. The percent deviance explained (PDE) by the BRT models for the calibration and cross-validation (CV) processes and the final number of predictors (Xi) is shown for Hā‘ena and Kaʻūpūlehu.
(DOCX)

S1 Fig Measured versus modeled nutrients for groundwater and coastal discharge at Ka‘ūpūlehu. (TIFF)

S2 Fig Response curves of the benthic indicators at Hā‘ena. (TIF)

S3 Fig Response curves of the herbivore fish indicators at Hā‘ena. (TIF)

S4 Fig Response curves of the piscivore fish indicators at Hā‘ena. (TIF)

S5 Fig Response curves of benthic indicators at Ka‘ūpūlehu. (TIF)

S6 Fig Response curves of herbivore fish indicators at Ka‘ūpūlehu. (TIF)

S7 Fig Response curves of piscivore fish indicators at Ka‘ūpūlehu. (TIF)

S8 Fig Observed versus predicted coral reef indicators at Hā‘ena. (TIF)

S9 Fig Observed versus predicted coral reef indicators at Ka‘ūpūlehu. (TIF)
