# Supplementary material for: A linked land-sea modeling framework to inform ridge-to-reef management in high oceanic islands
Source: PLoS One. 2018 Mar 14;13(3):e0193230. doi: 10.1371/journal.pone.0193230 (PMC5851582; doi:10.1371/journal.pone.0193230)
Supplement: S1 File — (PDF) [file pone.0193230.s001.pdf]

“I request permission for the open-access journal PLOS ONE to publish *the wave model image* under the Creative Commons Attribution License (CCAL) CC BY 4.0 (hcp://creativecommons.org/licenses/by/4.0/). Please be aware that this license allows unrestricted use and distribution, even commercially, by third parties. Please reply and provide explicit written permission to publish *the wave model image* under a CC BY license.”

Permission granted to use the wave model image.


SOEST
SCHOOL OF OCEAN AND EARTH SCIENCE AND TECHNOLOGY
UNIVERSITY OF HAWAI'I AT MĀNOA
www.soest.hawaii.edu

**Charles “Chip” Fletcher**
Associate Dean for Academic Affairs
Professor of Geology and Geophysics

office 808.956.2582 cell 808.294.0386
fletcher@soest.hawaii.edu

1680 East-West Road, POST 802
Honolulu, Hawai‘i USA 96822
www.soest.hawaii.edu/coasts/
