# Supplementary material for: A linked land-sea modeling framework to inform ridge-to-reef management in high oceanic islands
Source: PLoS One. 2018 Mar 14;13(3):e0193230. doi: 10.1371/journal.pone.0193230 (PMC5851582; doi:10.1371/journal.pone.0193230)
Supplement: S1 Table — Benthic (% cover) and fish biomass (g.m-1) coral reef indicators were derived from the coral reef surveys and used as response variables in the coral reef models. (DOCX) [file pone.0193230.s002.docx]

# S1 Table. Modeling framework response variables description.

| Type | Code | Metric | Ecological value | Reference |
| --- | --- | --- | --- | --- |
| Benthic indicators | CCA | Crustose coralline algae | CCA and corals are active reef builders, which can foster recovery, larvae recruitment, and provide habitat for reef fishes. CCA also stabilize the reef in high-wave environment. | [1–6] |
|  | COR | Coral cover |  |  |
|  | MAC | Macroalgae | Excessive benthic algae can be a sign of excessive nutrients or reduced herbivory, which can affect coral health through direct or indirect competition for space, as well as reducing fish larvae settlement | [6–10] |
|  | TUR | Turf algae |  |  |
| Fish indicators | BROW | Browsers | Surgeonfishes and most Parrotfishes graze on turf algae or macroalgae, which can help reverse coral-algal phase shifts and some Parrotfishes free space for CCA and coral larval settlement | [1] |
|  | GRDT | Grazers |  |  |
|  | SCEX | Scrapers |  |  |
|  | PISC | Piscivores | High abundance of piscivores can be a sign of a healthy and not overfished population | [11] |

Benthic (% cover) and fish biomass (g.m^-1^) coral reef indicators were derived from the coral reef surveys and used as response variables in the coral reef models.

# References

1. Green AL, Bellwood DR. Monitoring functional groups of herbivorous reef fishes as indicators of coral reef resilience : a practical guide for coral reef managers in the Asia Pacific region. IUCN; 2009.

2. Goreau TF. Calcium carbonate deposition by coralline algae and corals in relation to their roles as reef-builders. Ann N Y Acad Sci. 1963;109: 127–167.

3. Harrington L, Fabricius K, De’Ath G, Negri A. Recognition and selection of settlement substrata determine post-settlement survival in corals. Ecology. 2004;85: 3428–3437.

4. Price N. Habitat selection, facilitation, and biotic settlement cues affect distribution and performance of coral recruits in French Polynesia. Oecologia. 2010;163: 747–758. doi:10.1007/s00442-010-1578-4

5. Setchell WA. Biotic cementation in coral reefs. Proc Natl Acad Sci. 1930;16: 781–783.

6. Smith JE, Brainard R, Carter A, Grillo S, Edwards C, Harris J, et al. Re-evaluating the health of coral reef communities: baselines and evidence for human impacts across the central Pacific. Proc R Soc B. 2016;283: 20151985. doi:10.1098/rspb.2015.1985

7. Houk P, Benavente D, Iguel J, Johnson S, Okano R. Coral Reef Disturbance and Recovery Dynamics Differ across Gradients of Localized Stressors in the Mariana Islands. PLOS ONE. 2014;9: e105731. doi:10.1371/journal.pone.0105731

8. Littler MM, Littler DS, Brooks BL. Harmful algae on tropical coral reefs: Bottom-up eutrophication and top-down herbivory. Harmful Algae. 2006;5: 565–585. doi:10.1016/j.hal.2005.11.003

9. Smith JE, Hunter CL, Smith CM. The effects of top–down versus bottom–up control on benthic coral reef community structure. Oecologia. 2010;163: 497–507.

10. Vermeij MJ, Sandin SA. Density-dependent settlement and mortality structure the earliest life phases of a coral population. Ecology. 2008;89: 1994–2004.

11. Wilson SK, Fisher R, Pratchett MS, Graham N a. J, Dulvy NK, Turner RA, et al. Habitat degradation and fishing effects on the size structure of coral reef fish communities. Ecol Appl. 2010;20: 442–451. doi:10.1890/08-2205.1
