# Supplementary material for: A linked land-sea modeling framework to inform ridge-to-reef management in high oceanic islands
Source: PLoS One. 2018 Mar 14;13(3):e0193230. doi: 10.1371/journal.pone.0193230 (PMC5851582; doi:10.1371/journal.pone.0193230)
Supplement: S2 Table — (DOCX) [file pone.0193230.s003.docx]

# S2 Table. Fish species composition per functional groups.

| Function | Scientific name | Common name | Hawaiian name |
| --- | --- | --- | --- |
| Browsers | *Calotomus carolinus* | Stareye parrotfish | ponuhunuhu |
|  | *Naso lituratus* | Orangespine unicornfish | umaumalei |
|  | *Naso unicornis* | Bluespine unicornfish | kala |
| Grazers | *Acanthurus achilles* | Achilles Tang | paku'iku'i |
|  | *Acanthurus blochii* | Ringtail surgeonfish | pualu |
|  | *Acanthurus dussumieri* | Eyestripe surgeonfish | palani |
|  | *Acanthurus guttatus* | Whitespotted surgeonfish | api |
|  | *Acanthurus leucopareius* | Whitebar surgeonfish | maikoiko |
|  | *Acanthurus nigricans* | Goldrim surgeonfish |  |
|  | *Acanthurus nigrofuscus* | Brown surgeonfish | ma'i'i'i |
|  | *Acanthurus nigroris* | Bluelined surgeonfish | maiko |
|  | *Acanthurus olivaceus* | Orangeband surgeonfish | na'ena'e |
|  | *Acanthurus triostegus* | Convict surgeonfish | manini |
|  | *Acanthurus xanthopterus* | Yellowfin surgeonfish | pualu |
|  | *Ctenochaetus hawaiiensis* | Black surgeonfish |  |
|  | *Ctenochaetus strigosus* | Goldring surgeonfish | kole |
|  | *Zebrasoma flavescens* | Yellow tang | lau'ipala |
|  | *Zebrasoma veliferum* | Sailfin tang | mane'one'o |
| Scrapers | *Chlorurus perspicillatus* | Spectacled parrotfish | uhu uliuli |
|  | *Chlorurus spilurus* | Pacific bullethead parrotfish | uhu |
|  | *Scarus dubius* | Regal parrotfish | lauia |
|  | *Scarus psittacus* | Palenose parrotfish | uhu |
|  | *Scarus rubroviolaceus* | Ember parrotfish | palukaluka |
| Piscivores | *Caranx melampygus* | Bluefin trevally | omilu |
|  | *Parupeneus cyclostomus* | Blue goatfish | moano kea |
|  | *Scomberoides lysan* | Doublespotted queenfish | lai |
