# Supplementary material for: A linked land-sea modeling framework to inform ridge-to-reef management in high oceanic islands
Source: PLoS One. 2018 Mar 14;13(3):e0193230. doi: 10.1371/journal.pone.0193230 (PMC5851582; doi:10.1371/journal.pone.0193230)
Supplement: S3 Table — See Carlson and Wiegner [169] for more details on sample collection, processing, and analytical methods. (DOCX) [file pone.0193230.s004.docx]

# S3 Table. Coastal water quality data at Ka‘ūpūlehu.

| **Transect** | **Site** | **Distance to shore (m)** | **[N]**  **(mg.L^-1^)** | **[P]**  **(mg.L^-1^)** |
| --- | --- | --- | --- | --- |
| 1 | 0 | 0 | 13.3 | 0.2 |
| 1 | 1 | 5 | 12.53 | 0.33 |
| 1 | 2 | 10 | 11.27 | 0.23 |
| 1 | 3 | 20 | 7.7 | 0.1 |
| 1 | 4 | 25 | 7.1 | 0.13 |
| 1 | 5 | 50 | 6.33 | 0.1 |
| 1 | 6 | 75 | 5.37 | 0.1 |
| 1 | 7 | 100 | 5.63 | 0.1 |
| 2 | 8 | 0 | 17.03 | 0.27 |
| 2 | 9 | 5 | 19.33 | 0.27 |
| 2 | 10 | 10 | 19.27 | 0.17 |
| 2 | 11 | 20 | 15.7 | 0.3 |
| 2 | 12 | 25 | 14.67 | 0.2 |
| 2 | 13 | 50 | 9.5 | 0.23 |
| 2 | 14 | 75 | 4.8 | 0.1 |
| 2 | 15 | 100 | 3.47 | 0.1 |

See Carlson and Wiegner [1] for more details on sample collection, processing, and analytical methods.

**Reference**

1. Carlson KM, Wiegner TN. Effects of submarine groundwater discharge on bacterial growth efficiency in coastal Hawaiian waters. Aquat Microb Ecol. 2016;77: 167–181.
