# Supplementary material for: A linked land-sea modeling framework to inform ridge-to-reef management in high oceanic islands
Source: PLoS One. 2018 Mar 14;13(3):e0193230. doi: 10.1371/journal.pone.0193230 (PMC5851582; doi:10.1371/journal.pone.0193230)
Supplement: S4 Table — This table provides the hypothesized relationships between the drivers and coral reef indicators. (DOCX) [file pone.0193230.s005.docx]

# S4 Table. Modeling framework predictor variables description and processing methods.

| **Type** | **Code** | **Metric** | **Source** | **Description** | **Analytical tool** |
| --- | --- | --- | --- | --- | --- |
| Terrestrial drivers | H_2_O | Freshwater | Groundwater models | Proxy for salinity (.yr^-1^) | GIS-based models |
|  | N | Dissolved nitrogen | Groundwater models | Proxy for land-based source dissolved nitrogen (.yr^-1^) | GIS-based models |
|  | P | Dissolved phosphorus | Groundwater models | Proxy for land-based source dissolved phosphorus (.yr^-1^) | GIS-based models |
| Marine driver (Wave) | wav | Wave power | SWAN wave model^a^ | Wave power (kW.m^-1^) | [1] |
| Marine drivers (Geography) | Depth | Depth | Bathymetry^b^ | Average depth (m) | ArcGIS Spatial Analyst tools [2] |
|  | dist2shore | Distance to shore | Coastline^c^ | Distance to nearest land (m) | ArcGIS Spatial Analyst Euclidean Distance tool [2] |
| Marine drivers (Habitat topography) | bpi | Bathymetric position index (60m, 240m) | Bathymetry^b^ | Mean values indicate a location’s position relative to the surrounding area; values can be positive (ridges), negative (valleys), or zero (flat or constant slope) | Benthic Terrain Modeler tool [3] |
|  | slp | Slope  (60m, 240m) | Bathymetry^b^ | Maximum rate of change from a cell to its neighbors | ArcGIS Slope tool [2]  ArcGIS Focal Statistics tool [2] |
| Marine drivers (Habitat exposure) | asp_sd | Surface aspect  (standard deviation) | Bathymetry^b^ | Slope direction (degrees) | ArcGIS Aspect tool [2] |
|  | asp_sin | Sine aspect | Bathymetry^b^ | Sine of slope direction (derived from transforming the mean aspect into “eastness”) (degrees) | ArcGIS Spatial Analyst tools (sine function) [2] |
|  | asp_cos | Cosine aspect | Bathymetry^b^ | Cosine of slope direction (derived from transforming the mean aspect into “northness”) (degrees) | ArcGIS Spatial Analyst tools (cosine function) [2] |
| Marine drivers (Habitat complexity) | curv_pro | Profile curvature (mean) | Bathymetry^b^ | Curvature values can be + (concave), - (convex), or 0 (flat). A proxy for spur and groove effects on water flow. | DEM Surface Tools Curvature tool [4] |
|  | curv_plan | Planar curvature (mean) | Bathymetry^b^ | Curvature values can be – (concave) to + (convex), or 0 (flat) (mean). A proxy for spur and groove effects on water flow. | DEM Surface Tools Curvature tool [4] |
|  | rug | Rugosity | Bathymetry^b^ | Value range from 1 (flat) to infinity. | DEM Surface Tools Curvature tool [4] |
| Benthic community | CCA | Crustose coralline algae | Coral reef model | Spatially-explicit predicted % cover | Coral reef model predictions |
|  | COR | Coral cover | Coral reef model | Spatially-explicit predicted % cover | Coral reef model predictions |
|  | MAC | Macroalgae | Coral reef model | Spatially-explicit predicted % cover | Coral reef model predictions |
|  | TUR | Turf algae | Coral reef model | Spatially-explicit predicted % cover | Coral reef model predictions |

This table provides a description of all the predictor variables modeled in the coral reef models. Each metric is classified by type (terrestrial drivers or marine drivers) and assigned a code for modeling. The table below indicates the data source and analytical tool used to generate each metric at 60 m resolution. Refer to Stamoulis & Delevaux et al. [5] for more details on processing methods.

^a^ SWAN hindcast wave model at 500 m native resolution [1]

^b^ Bathymetry synthesis at 5 m native resolution [6]

^c^ Coastline [7]

**References**

1. Stopa JE, Filipot J-F, Li N, Cheung KF, Chen Y-L, Vega L. Wave energy resources along the Hawaiian Island chain. Renew Energy. 2013;55: 305–321. doi:10.1016/j.renene.2012.12.030

2. ESRI. ArcGIS Desktop: Release 10. Environmental Systems Research Institute. [Internet]. Redlands, CA.; 2011. Available: http://www. esri.com/

3. Wright DJ, Lundblad ER, Larkin EM, Rinehart RW, Murphy J, Cary-Kothera L, et al. ArcGIS Benthic Terrain Modeler [a collection of tools used with bathymetric data sets to examine the deepwater benthic environment]. Or State Univ Davey Jones’ Locker Seafloor MappingMarine GIS Lab NOAA Coast Serv Cent. 2005;

4. Jenness J. DEM surface tools for ArcGIS. Jenness Enterp. 2013; 1–96.

5. Stamoulis KA, Delevaux JMS, Williams I, Poti M, Costa B, Kendall MS, et al. Seascape models reveal places to focus coral reef fisheries management. Ecol Appl. submitted;

6. HMRG. Bathymetry [Internet]. University of Hawai‘i at Manoa, HI, USA: Hawai‘i Mapping Research Group, School of Ocean and Earth Science and Technology; 2015. Available: http://www.soest.hawaii.edu/HMRG/cms/

7. OP. Coastlines for the main hawaiian islands [Internet]. Honolulu, Hawaii, USA: Hawaii Office of Planning; 2000. Available: http://planning.hawaii.gov/gis/download-gis-data/
