# Supplementary material for: A linked land-sea modeling framework to inform ridge-to-reef management in high oceanic islands
Source: PLoS One. 2018 Mar 14;13(3):e0193230. doi: 10.1371/journal.pone.0193230 (PMC5851582; doi:10.1371/journal.pone.0193230)
Supplement: S5 Table — This table provides a description of all the predictor variables modeled in the coral reef models. Each metric is classified by type (terrestrial drivers or marine drivers) and assigned a code for modeling. The table below indicates the data source and analytical tool used to generate each metric. Refer to Stamoulis & Delevaux et al. [110] for more details on processing methods. (DOCX) [file pone.0193230.s006.docx]

# S5 Table. Response variables and drivers’ relationships.

| **Drivers** | **Indicators** | **Hypothesized relationships** | **Reference** |
| --- | --- | --- | --- |
| Freshwater | Reef builders | Decreased salinity can inhibit CCA and coral growth | [1,2] |
| Nutrients | Benthic algae | Increased nutrients can promote algae growth |  |
| Wave power | CCA | Increased wave power can promote CCA growth | [3–5] |
|  | Coral | Increased wave power can inhibit coral growth |  |
|  | Resource fishes | Higher resource fish biomass is expected in areas with higher wave power which creates protection from fishing pressure |  |
| Geography (Depth and Distance to shore) | Reef builders | Reef builders abundance is higher in offshore and deeper waters (as long as light conditions allow for photosynthesis) | [3,6,7] |
|  | Benthic algae | Benthic algae abundance is higher nearshore and in shallow waters. |  |
|  | Resource fishes | Higher resource fish biomass is expected in offshore and deeper waters protected from fishing pressure |  |
| Habitat topography (reef flats and slopes) | Reef builders | Reef builders abundance is higher along reef slopes | [8,9] |
|  | Benthic algae | Benthic algae abundance is higher on reef flats |  |
|  | Resource fishes | Higher resource fish biomass is expected along the reef slopes |  |
| Habitat exposure | Reef builders | Reef builders abundance is higher in exposed areas where water is well mixed | [10,11] |
|  | Benthic algae | Benthic algae abundance is higher in sheltered areas with poor mixing |  |
| Habitat complexity | Reef builders | Reef builders abundance is higher in areas with higher complexity | [12,13] |
|  | Benthic algae | Benthic algae abundance is higher in areas with lower complexity |  |
|  | Resource fishes | Higher resource fish biomass is expected in areas with higher complexity |  |
| Reef builders | Resource fishes | Higher resource fish biomass is expected in areas with high reef builders abundance | [12] |
| Benthic algae | Herbivores | Higher resource fish biomass is expected in areas with high turf algae cover | [14] |

This table provides the hypothesized relationships between the drivers and coral reef indicators.

**References**

1. Smith JE, Hunter CL, Smith CM. The effects of top–down versus bottom–up control on benthic coral reef community structure. Oecologia. 2010;163: 497–507.

2. Littler MM, Littler DS, Brooks BL. Harmful algae on tropical coral reefs: Bottom-up eutrophication and top-down herbivory. Harmful Algae. 2006;5: 565–585. doi:10.1016/j.hal.2005.11.003

3. Dollar SJ. Wave stress and coral community structure in Hawaii. Coral Reefs. 1982;1: 71–81. doi:10.1007/BF00301688

4. Grigg RW. Holocene coral reef accretion in Hawaii: a function of wave exposure and sea level history. Coral Reefs. 1998;17: 263–272. doi:10.1007/s003380050127

5. Friedlander AM, Brown EK, Jokiel PL, Smith WR, Rodgers KS. Effects of habitat, wave exposure, and marine protected area status on coral reef fish assemblages in the Hawaiian archipelago. Coral Reefs. 2003;22: 291–305. doi:10.1007/s00338-003-0317-2

6. Jouffray J-B, Nyström M, Norström AV, Williams ID, Wedding LM, Kittinger JN, et al. Identifying multiple coral reef regimes and their drivers across the Hawaiian archipelago. Philos Trans R Soc B Biol Sci. 2015;370: 20130268. doi:10.1098/rstb.2013.0268

7. Fabricius K, De’ath G. Environmental factors associated with the spatial distribution of crustose coralline algae on the Great Barrier Reef. Coral Reefs. 2001;19: 303–309. doi:10.1007/s003380000120

8. Pittman SJ, Brown KA. Multi-Scale Approach for Predicting Fish Species Distributions across Coral Reef Seascapes. PLOS ONE. 2011;6: e20583. doi:10.1371/journal.pone.0020583

9. Stamoulis KA, Poti M, Delevaux JMS, Donovan MK, Friedlander A, Kendall MS. Chapter 4: Fishes - Reef Fish. Marine Biogeographic Assessment of the Main Hawaiian Islands. B.M. Costa and M.S. Kendall. Bureau of Ocean Energy Management and National Oceanic and Atmospheric Administration. OCS Study BOEM 2016-035 and NOAA Technical Memorandum NOS NCCOS 214. 359 pp.; 2016. pp. 156–196.

10. Franklin EC, Jokiel PL, Donahue MJ. Predictive modeling of coral distribution and abundance in the Hawaiian Islands. Mar Ecol Prog Ser. 2013;481: 121–132.

11. Knudby A, Jupiter S, Roelfsema C, Lyons M, Phinn S. Mapping Coral Reef Resilience Indicators Using Field and Remotely Sensed Data. Remote Sens. 2013;5: 1311–1334. doi:10.3390/rs5031311

12. Friedlander AM, Parrish JD. Habitat characteristics affecting fish assemblages on a Hawaiian coral reef. J Exp Mar Biol Ecol. 1998;224: 1–30. doi:10.1016/S0022-0981(97)00164-0

13. Darling ES, Graham NA, Januchowski-Hartley FA, Nash KL, Pratchett MS, Wilson SK. Relationships between structural complexity, coral traits, and reef fish assemblages. Coral Reefs. 2017; 1–15.

14. Green AL, Bellwood DR. Monitoring functional groups of herbivorous reef fishes as indicators of coral reef resilience : a practical guide for coral reef managers in the Asia Pacific region. IUCN; 2009.
