# Supplementary material for: A linked land-sea modeling framework to inform ridge-to-reef management in high oceanic islands
Source: PLoS One. 2018 Mar 14;13(3):e0193230. doi: 10.1371/journal.pone.0193230 (PMC5851582; doi:10.1371/journal.pone.0193230)
Supplement: S6 Table — The percent deviance explained (PDE) by the BRT models for the calibration and cross-validation (CV) processes and the final number of predictors (Xi) is shown for Hā‘ena and Kaʻūpūlehu. (DOCX) [file pone.0193230.s007.docx]

# S6 Table. Coral reef predictive model performance per indicator.

| **Reef indicators** | **Hā‘ena** | | | **Ka‘ūpūlehu** | | |
| --- | --- | --- | --- | --- | --- | --- |
|  | **PDE (%)** | **CV PDE (%)** | **X_i_** | **PDE (%)** | **CV PDE (%)** | **X_i_** |
| CCA | 74 | 51 | 4 | 22 | 5 | 4 |
| Corals | 67 | 47 | 4 | 60 | 26 | 4 |
| Macroalgae | 50 | 27 | 7 | 28 | 7 | 4 |
| Turf algae | 44 | 10 | 6 | 33 | 10 | 6 |
| Browsers | 34 | 12 | 5 | 21 | 5 | 6 |
| Grazers | 49 | 25 | 7 | 50 | 16 | 6 |
| Scrapers | 41 | 10 | 6 | 50 | 20 | 5 |
| Piscivores | 41 | 10 | 6 | 32 | 10 | 7 |

The percent deviance explained (PDE) by the BRT models for the calibration and cross-validation (CV) processes and the final number of drivers (X**_i_**) is shown for Hā‘ena and Kaʻūpūlehu.
