# Supplementary figures and images for: A linked land-sea modeling framework to inform ridge-to-reef management in high oceanic islands
Source: PLoS One. 2018 Mar 14;13(3):e0193230. doi: 10.1371/journal.pone.0193230 (PMC5851582; doi:10.1371/journal.pone.0193230)

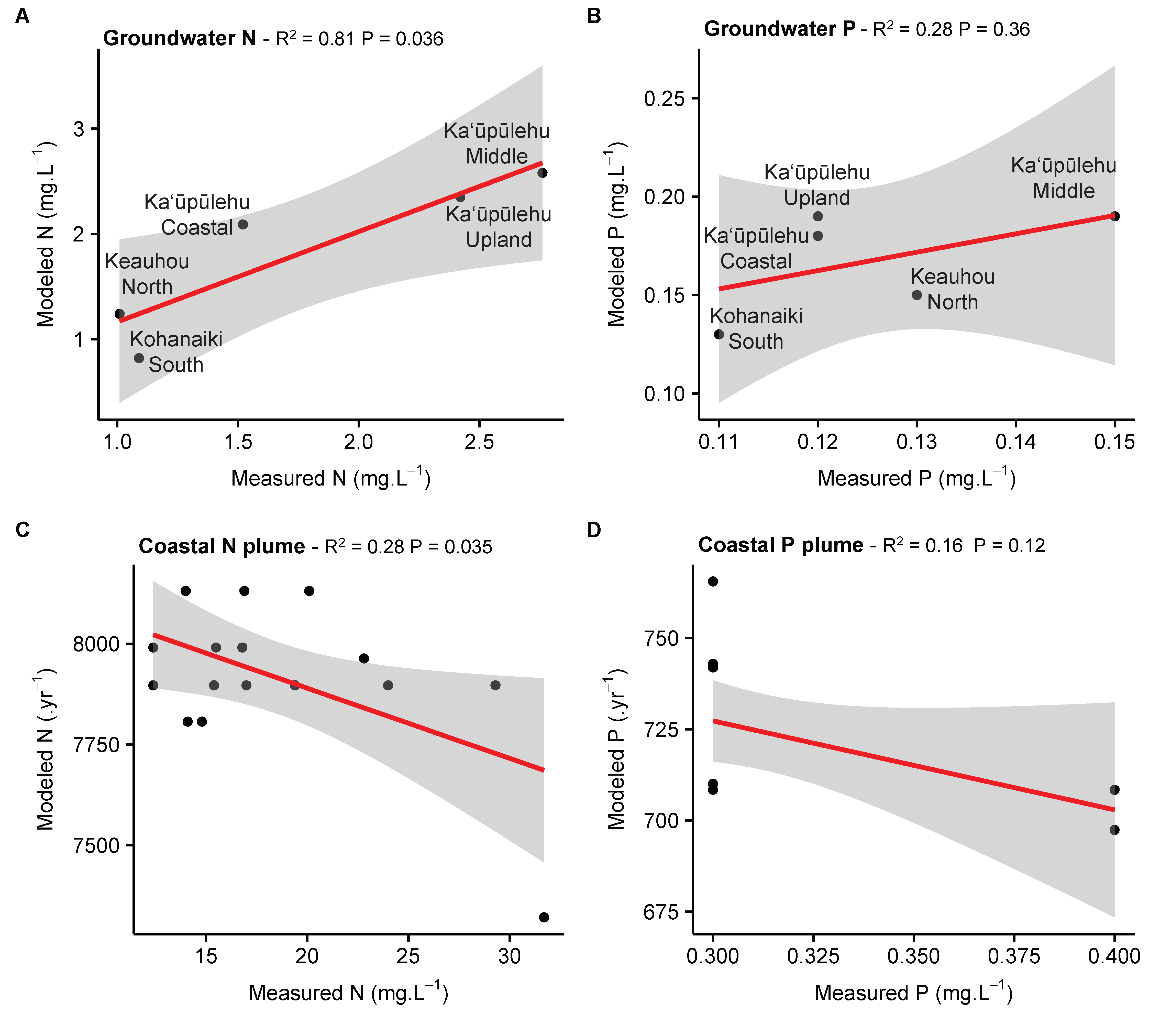

Supplement: S1 Fig — (TIFF) [file pone.0193230.s008.tiff]

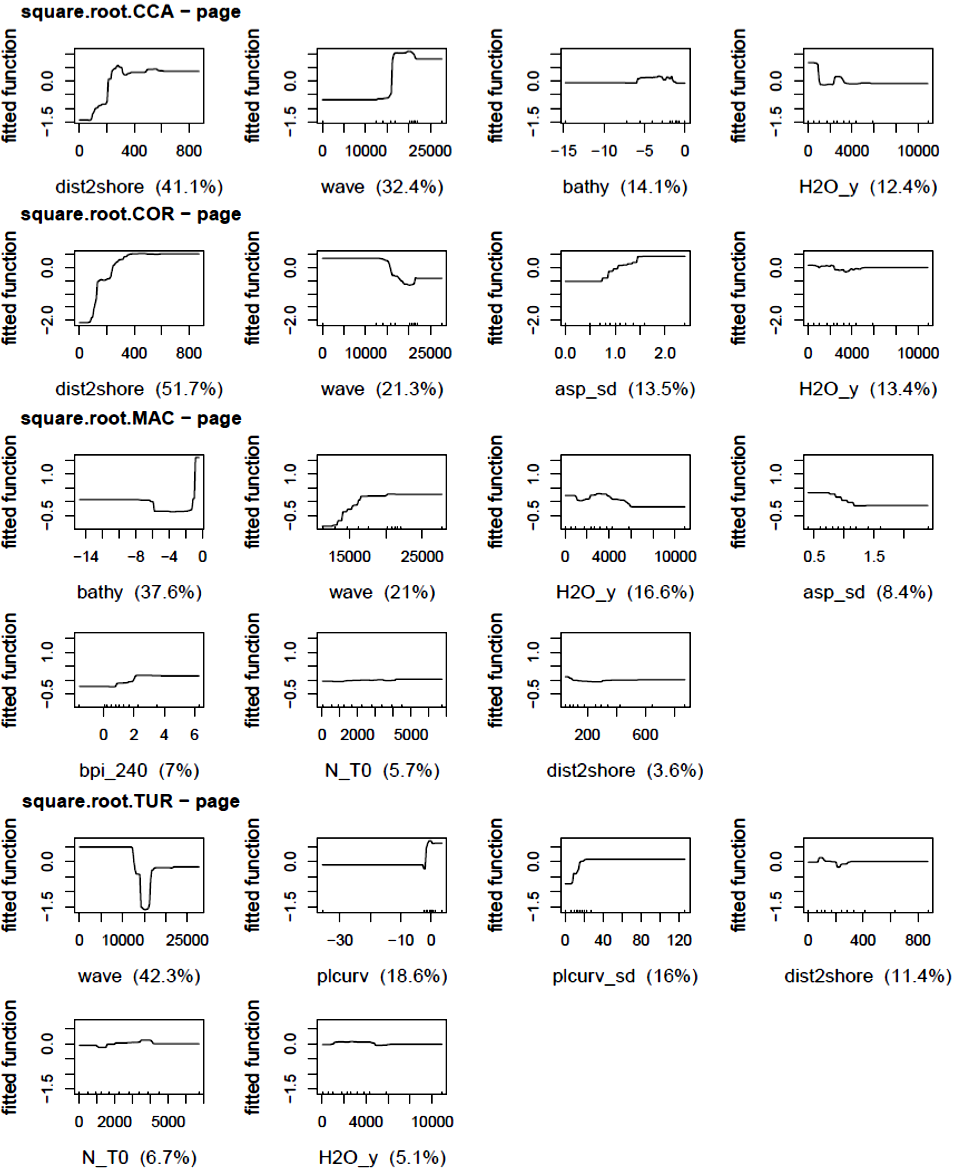

Supplement: S2 Fig — (TIF) [file pone.0193230.s009.tif]

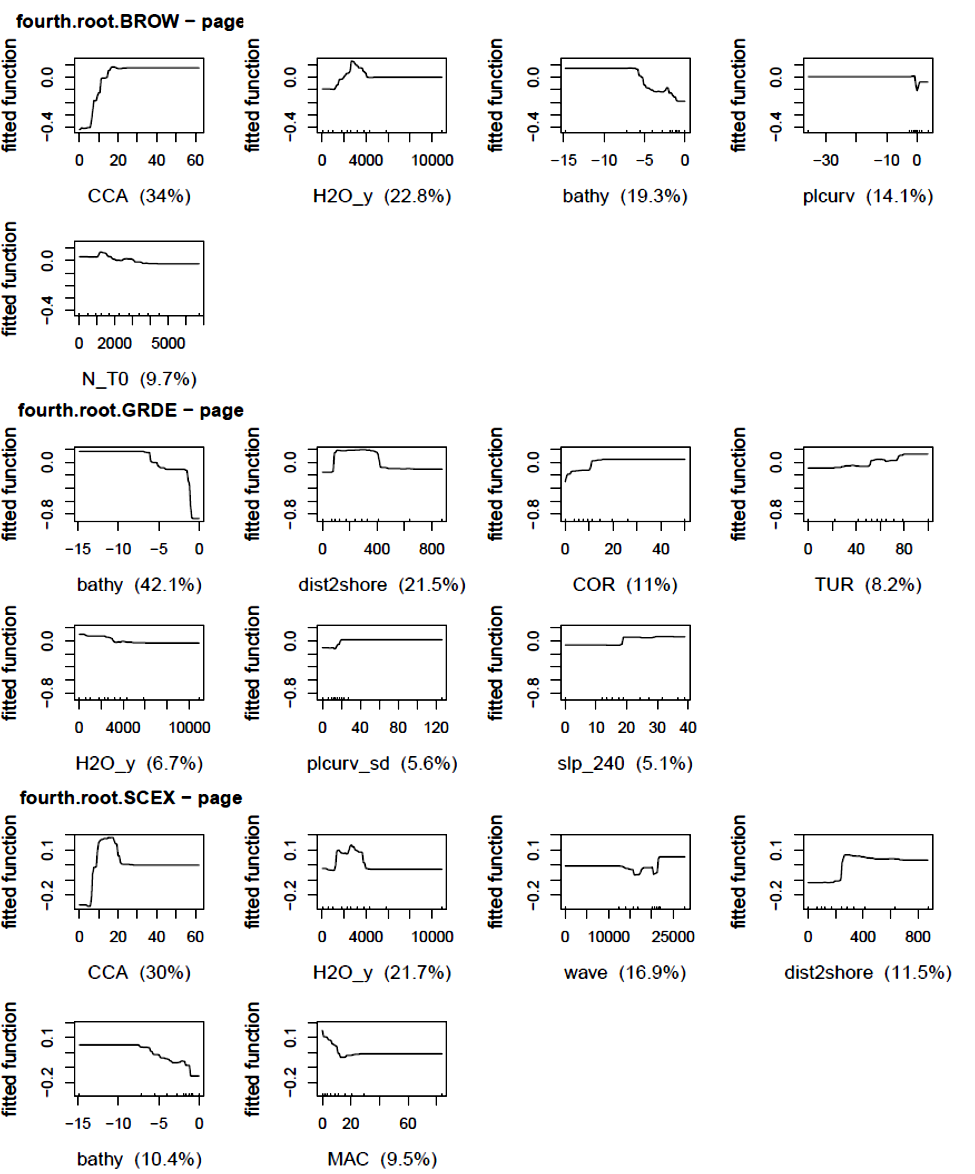

Supplement: S3 Fig — (TIF) [file pone.0193230.s010.tif]

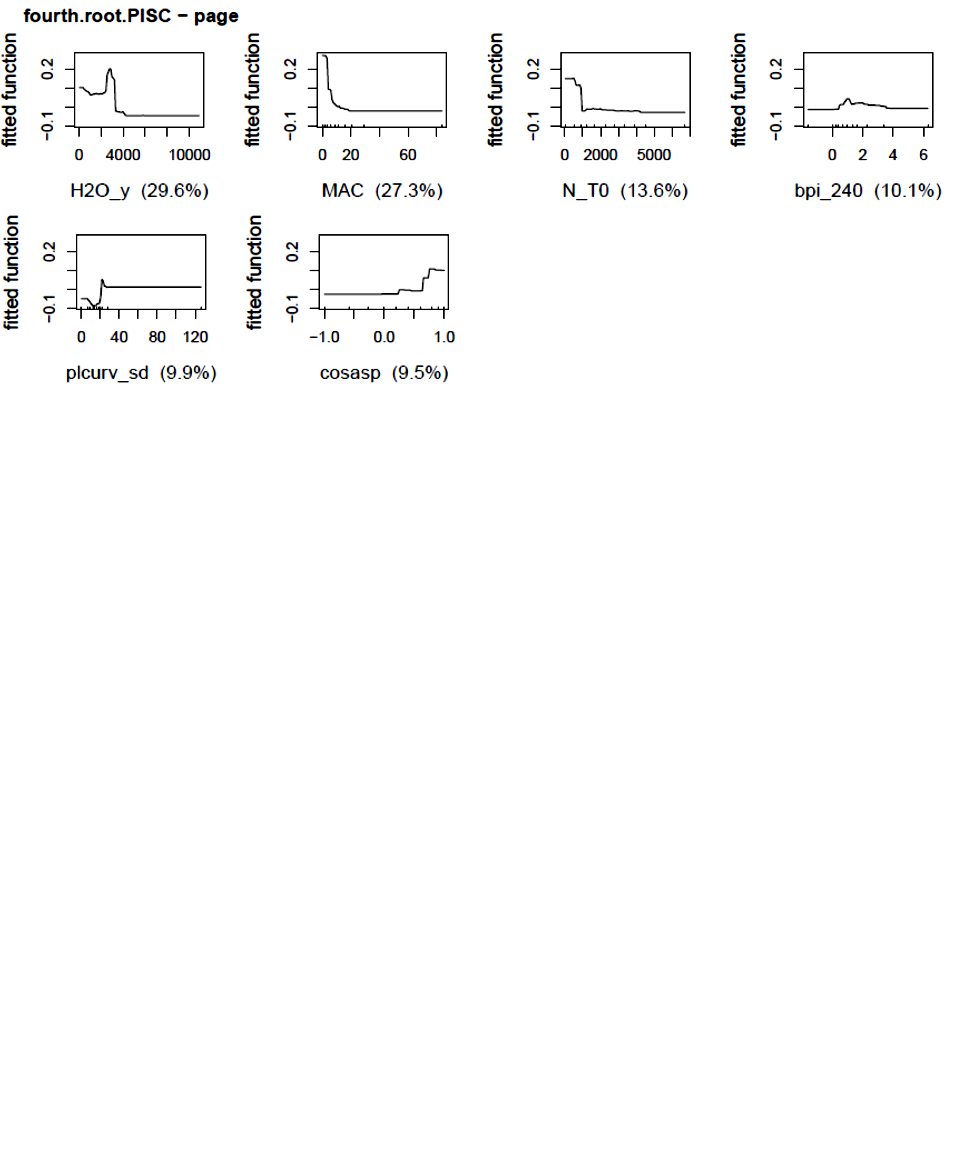

Supplement: S4 Fig — (TIF) [file pone.0193230.s011.tif]

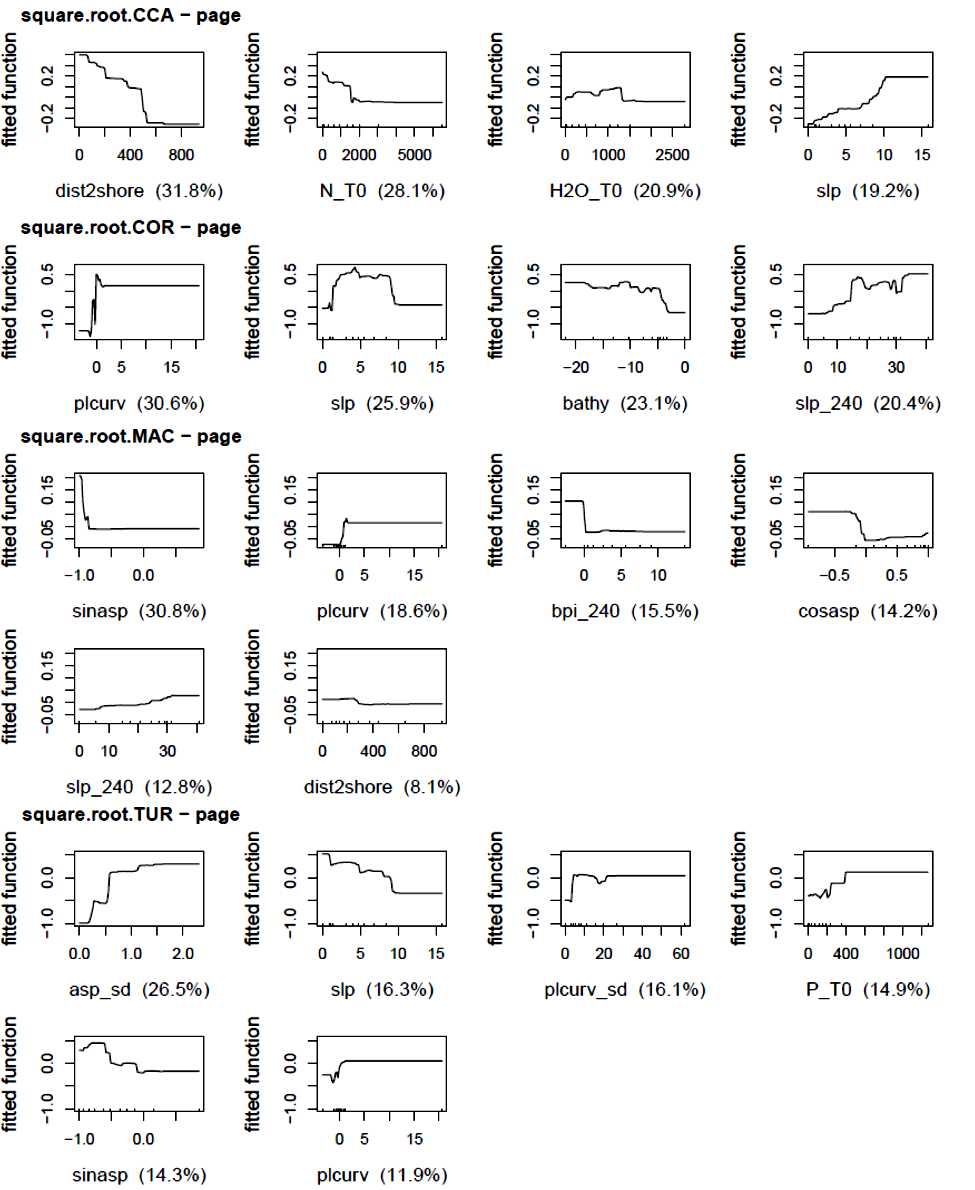

Supplement: S5 Fig — (TIF) [file pone.0193230.s012.tif]

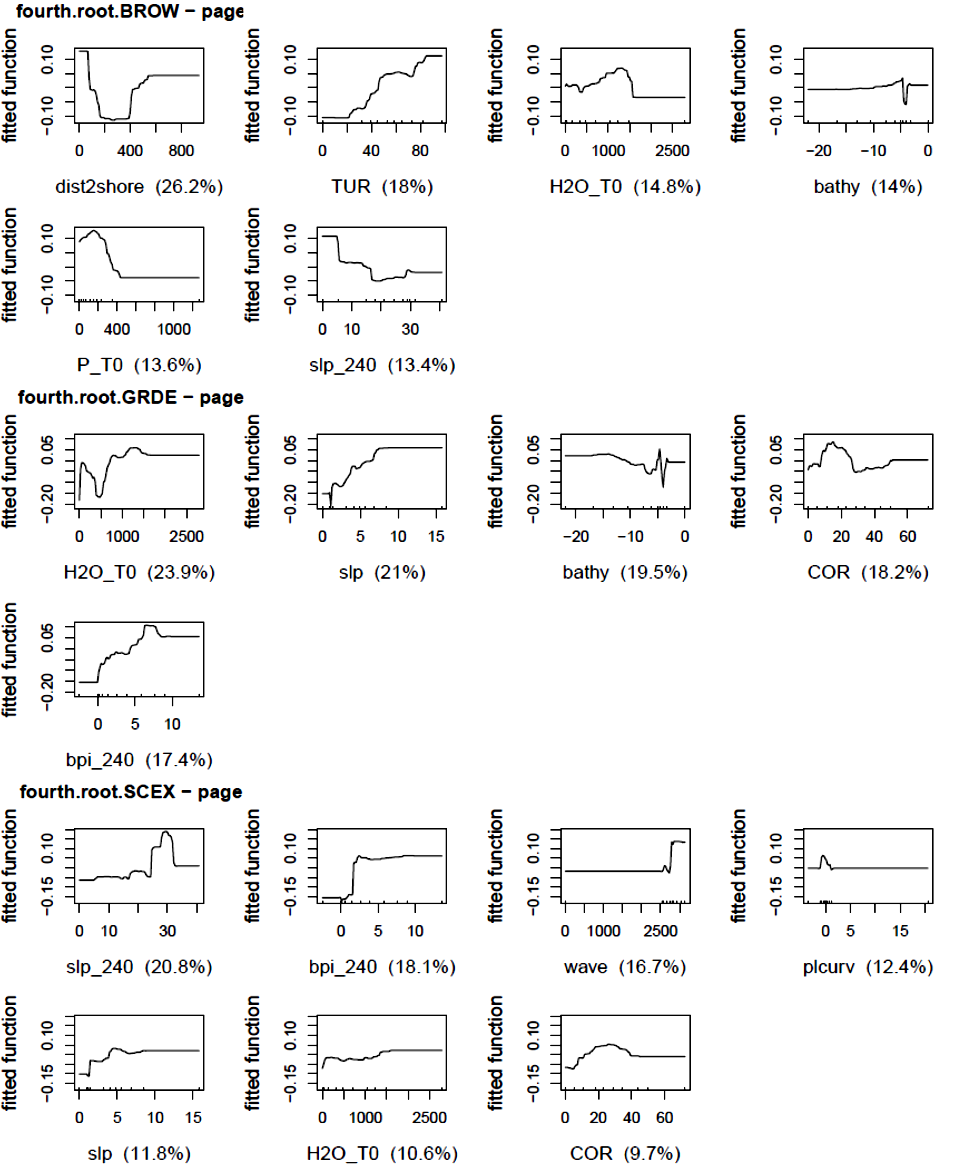

Supplement: S6 Fig — (TIF) [file pone.0193230.s013.tif]

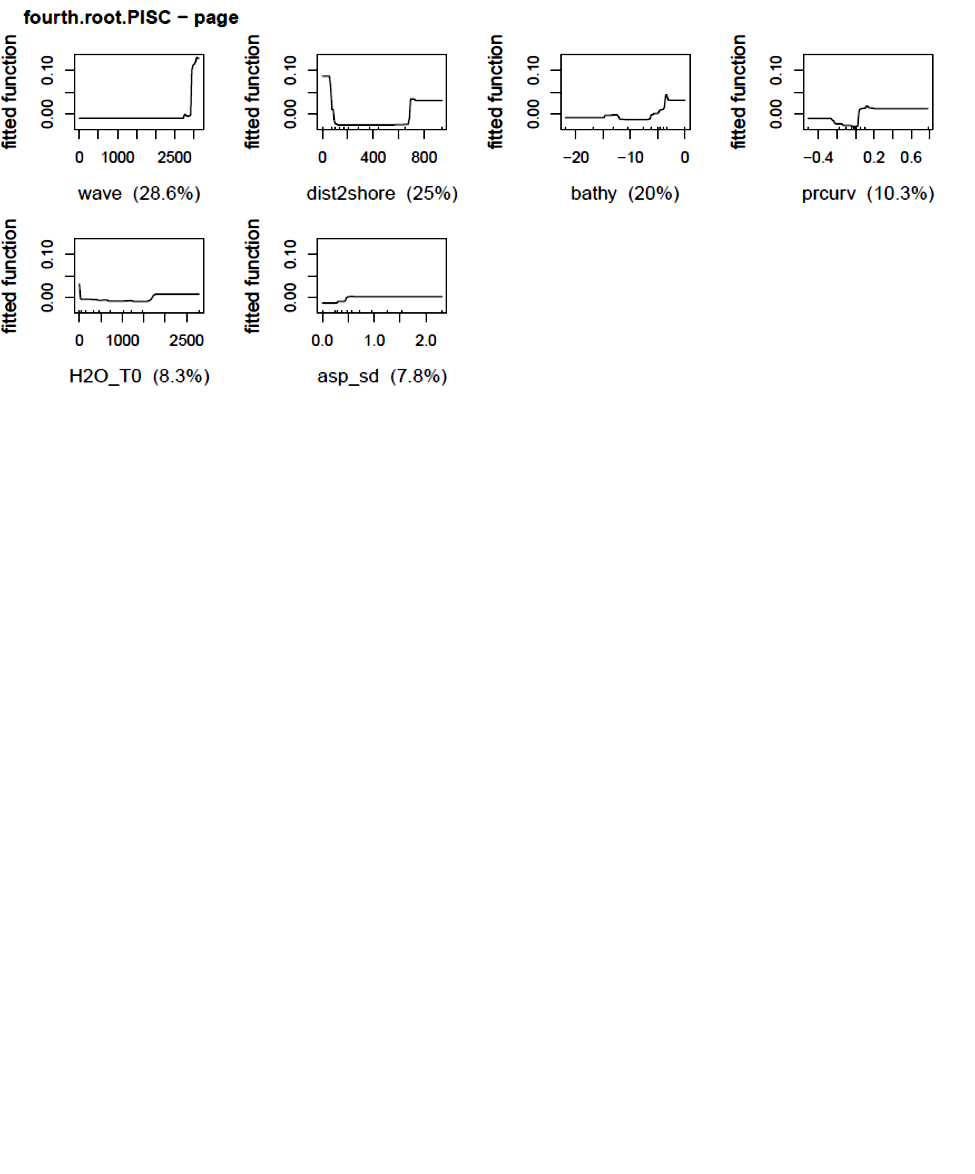

Supplement: S7 Fig — (TIF) [file pone.0193230.s014.tif]

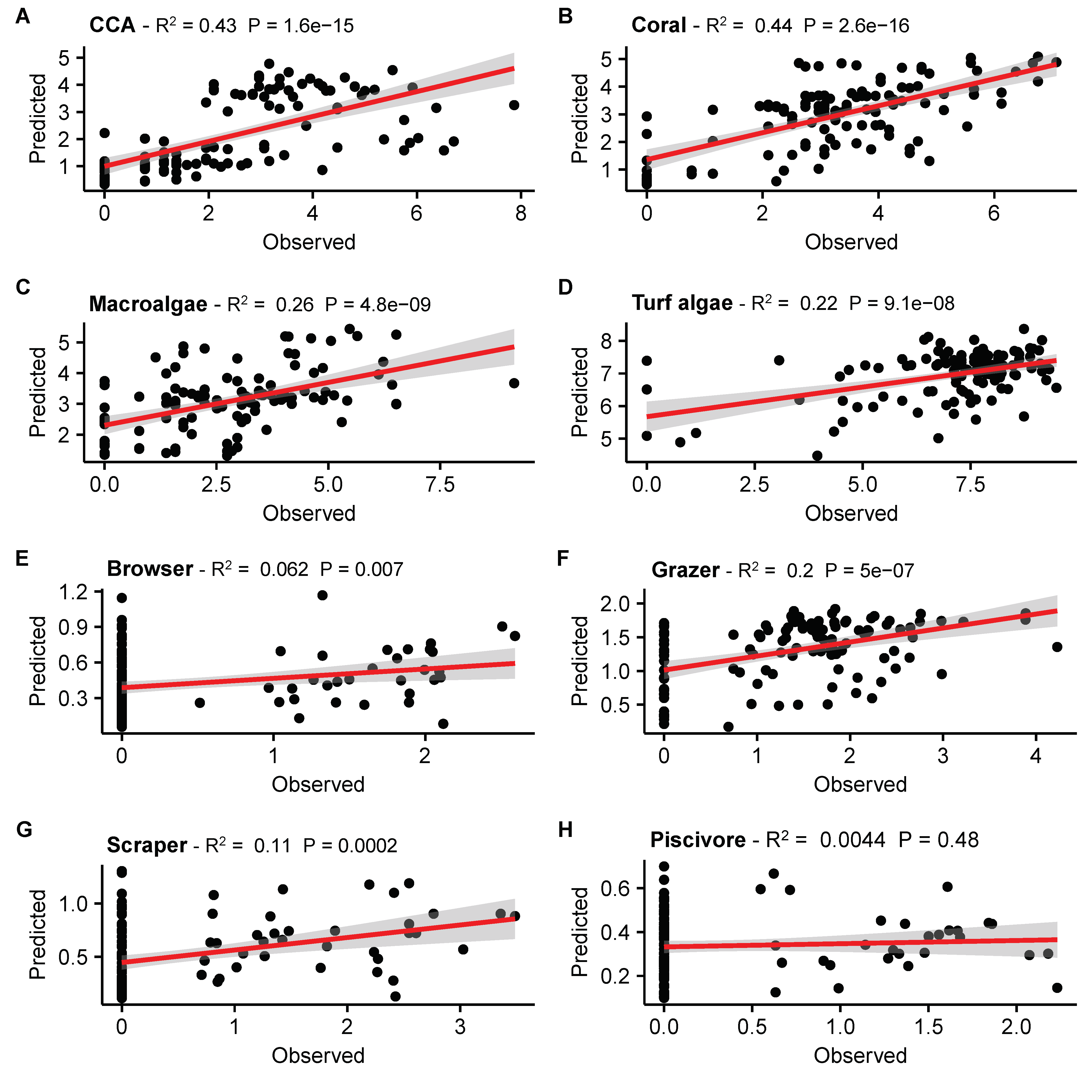

Supplement: S8 Fig — (TIF) [file pone.0193230.s015.tif]

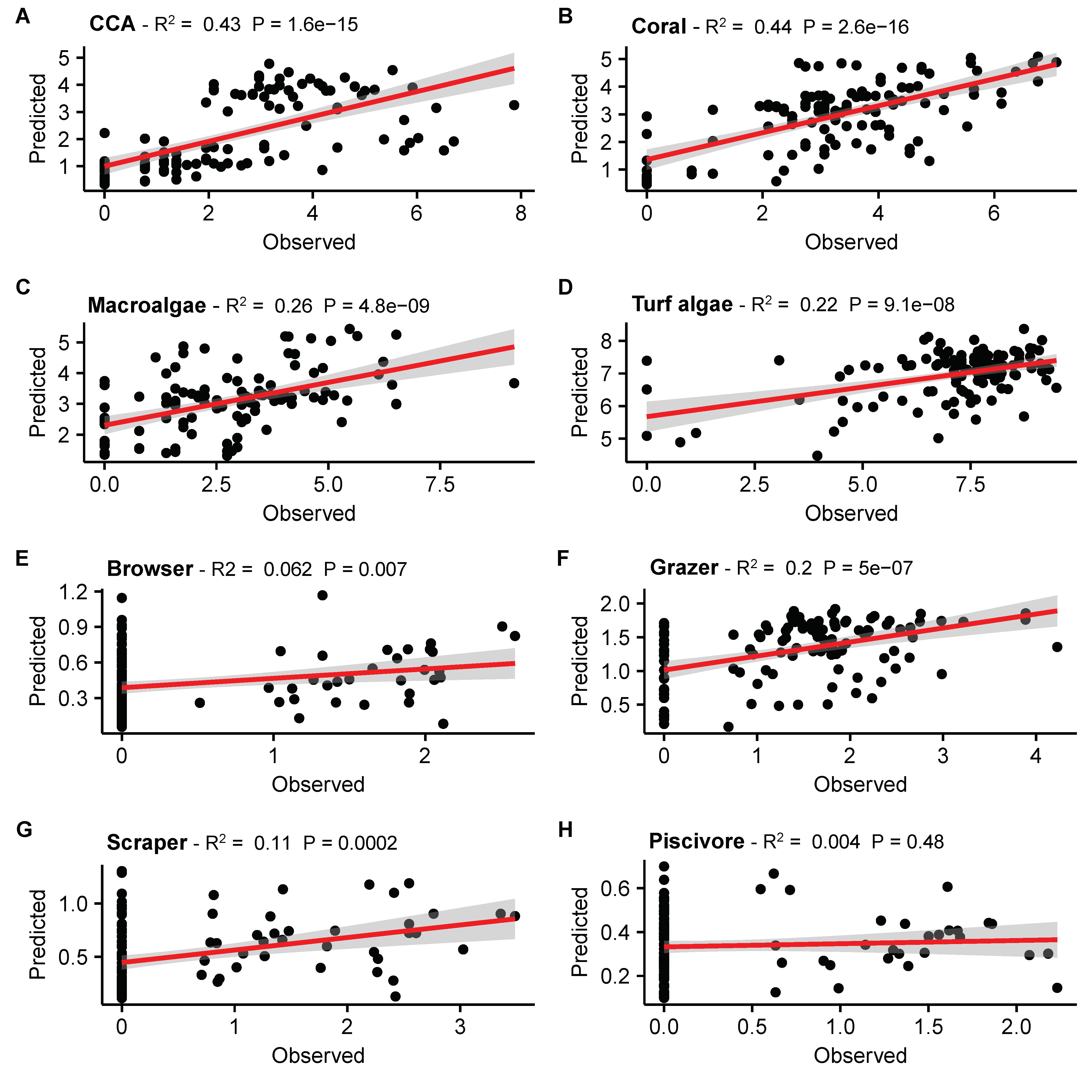

Supplement: S9 Fig — (TIF) [file pone.0193230.s016.tif]
